# Monocyte subpopulation profiling indicates CDK6-derived cell differentiation and identifies subpopulation-specific miRNA expression sets in acute and stable coronary artery disease

**DOI:** 10.1038/s41598-022-08600-7

**Published:** 2022-04-04

**Authors:** Anika Witten, Leonie Martens, Ann-Christin Schäfer, Christian Troidl, Sabine Pankuweit, Ann-Kathrin Vlacil, Raghav Oberoi, Bernhard Schieffer, Karsten Grote, Monika Stoll, Birgit Markus

**Affiliations:** 1 Genetic Epidemiology, Institute for Human Genetics, Westfälische Wilhelms-University, Domagkstraße 3, 48149 Münster, Germany; 2 Core Facility Genomik, Medical Faculty Münster, Westfälische Wilhelms-University, Münster, Germany; 3 Department of Cardiology and Angiology, Philipps-University Marburg, Marburg, Germany; 4 Department of Experimental Cardiology, Justus-Liebig-University, Giessen, Germany; 5 Department of Biochemistry, Cardiovascular Research Institute Maastricht, Maastricht University, Maastricht, The Netherlands

**Keywords:** miRNA in immune cells, Cardiovascular biology

## Abstract

Coronary artery disease (CAD) is a long-lasting inflammatory disease characterized by monocyte migration into the vessel wall leading to clinical events like myocardial infarction (MI). However, the role of monocyte subsets, especially their miRNA-driven differentiation in this scenario is still in its infancy. Here, we characterized monocyte subsets in controls and disease phenotypes of CAD and MI patients using flow cytometry and miRNA and mRNA expression profiling using RNA sequencing. We observed major differences in the miRNA profiles between the classical (CD14^++^CD16^−^) and nonclassical (CD14^+^CD16^++^) monocyte subsets irrespective of the disease phenotype suggesting the Cyclin-dependent Kinase 6 (CDK6) to be an important player in monocyte maturation. Between control and MI patients, we found a set of miRNAs to be differentially expressed in the nonclassical monocytes and targeting CCND2 (Cyclin D2) that is able to enhance myocardial repair. Interestingly, miRNAs as miR-125b playing a role in vascular calcification were differentially expressed in the classical subset in patients suffering from CAD and not MI in comparison to control samples. In conclusion, our study describes specific peculiarities of monocyte subset miRNA expression in control and diseased samples and provides basis to further functional analysis and to identify new cardiovascular disease treatment targets.

## Introduction

Monocytes, dendritic cells and macrophages are the three major members of a family of myeloid cells. While macrophages can mature from monocytes, blood monocytes and dendritic cells arise from distinct adult hematopoietic stem cell precursors in the bone marrow^1,2^. Monocytes are not restricted to function as a source for recruited macrophages or dendritic cells during inflammation; the circulating and dynamic monocyte population plays an important role in modulating innate immune response and initiating adaptive immunity^1,3^. Therefore, monocytes have become a major area of interest in the quest to disentangle the mechanisms underlying a disease as coronary artery disease (CAD) driven by chronic inflammation. Circulation monocytes comprise multiple subsets, which can be characterized by the expression of markers like the CC-chemokine receptor-2 (CCR2), their response to lipopolysaccharide (LPS) in vitro, their morphology and function^4^. Since 2010, the monocyte population is divided into three subsets characterized by the cell-surface markers CD14 and CD16: classical monocytes (CD14^++^CD16^−^), intermediate monocytes (CD14^++^CD16^+^) and nonclassical monocytes (CD14^+^CD16^++^)^5,6^. About 85% of monocytes consist of classical monocytes; this prevalent subset has phagocytic properties and is rapidly recruited to sites of infection or tissue damage. The expression of the chemokine receptor CCR2 which mediates monocyte tissue recruitment is mostly restricted to this subset^4^. Intermediate monocyte represent a small percentage exhibiting phagocytic and inflammatory attributes^2^. The nonclassical monocytes perform a patrolling function along the vascular endothelium and are involved in tissue repair. Both CD16-positive monocyte subsets have pro-inflammatory properties^3^. In vitro, for instance, CD16-positive monocytes produce higher amounts of TNF (tumor necrosis factor) in response to toll-like receptor (TLR) stimulation in comparison to classical monocytes. In vivo, the CD16-positive subpopulation expands under inflammatory conditions such as CAD and infection^7^.

Monocytes exhibit diverse functions during chronic vascular inflammation and after myocardial infarction (MI). Monocytes are the primary inflammatory cells that enter early atherosclerotic lesions, and it has been shown that amount and composition of invading monocytes are crucial for further plaque development^8,9^. It is well known, that circulating total monocytes are increased during atherosclerosis as well as after MI^8–11^ and the composition of monocyte subsets changes dynamically during disease progression^12^. Intermediate monocytes are expanded in CAD, they expand in patients with active inflammation, while the classical subset is decreased. Rogacev et al. therefore postulate the intermediate subpopulation as an independent biomarker to predict cardiovascular events^13^. Several expression profiling studies for mRNA transcripts and miRNAs have been published in the past decade, defining the subsets mostly in control samples. Wong and colleagues reported a relationship between the intermediate and nonclassical monocyte subsets, as well as the transitional nature of the intermediate population. This study is supported by a regulome analysis of the three populations where subset-specific promoter and enhancer landscapes were defined^14^. Zawada et al. performed a first genome-wide miRNA profiling to characterize monocyte subsets and detected miRNAs such as miR-6087 and miR-150-5p mostly expressed by intermediated monocytes, providing further evidence for the transitional state of this monocyte population^15^.

Nevertheless, studies published so far did not take account of transcript and miRNA expression profiling in control and diseased samples in parallel. Therefore, we conducted and report flow cytometric analysis and genome-wide transcriptional profiling for miRNA and mRNA species and their correlation within the three monocyte subsets derived from patients suffering from first acute MI, stable without any history of cardiovascular disease.

## Results

### Study outline and flow cytometric analysis of monocyte subpopulations from patients with different stages of CAD

**Figure 1:**
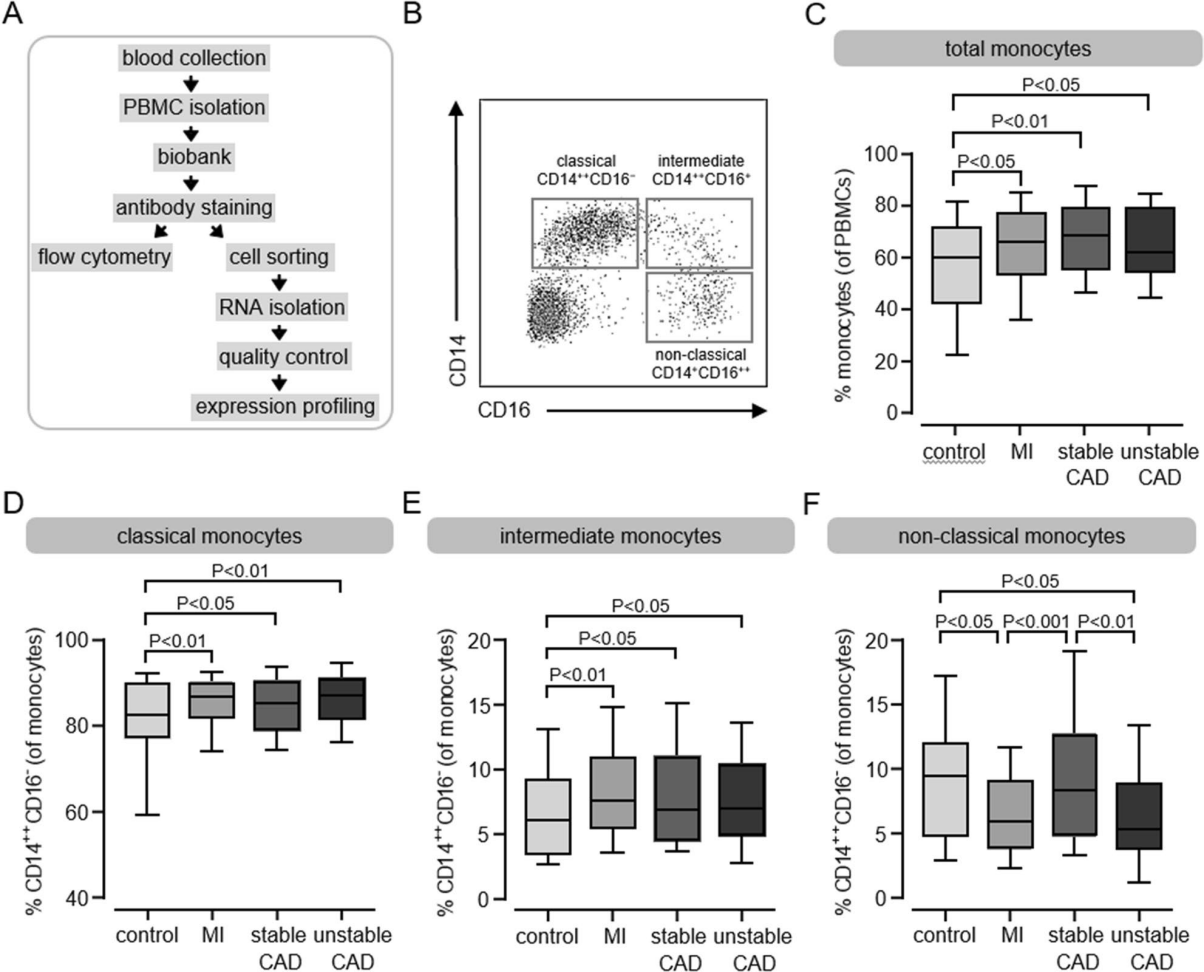
Analysis of monocyte subpopulations in patients with different stages of MI and CAD. (**A**) Flow chart of the experimental setup. (**B**) Sorting gates for the identification of human circulating blood monocyte subpopulations based on their relative CD14 and CD16 expression by flow cytometry and cell sorting. (**C**) Quantification of total monocytes, classical monocytes (**D**), intermediate monocytes (**E**) and nonclassical monocytes (**F**) in control subjects (n = 61) and in patients with MI (n = 110), stable CAD (n = 69) and unstable CAD (n = 65) by flow cytometry. Box plots with median and 25th/75th percentiles (boxes) and 10th/90th percentiles (whiskers).

In total, 244 CAD patients were included and grouped as follows: 110 patients having a first acute MI, 69 patients with stable CAD, 65 patients with unstable CAD and 61 control patients without any history of cardiovascular disease. Characteristics of all samples are given in Supplementary Table S1, sample handling is given in Fig. 1A.

By negative selection and based on their CD14 and CD16 expression, circulating subpopulations were identified and distinguished by flow cytometry using an established gating strategy^16^ (Fig. 1B and Supplementary Figure S1). Based on the CD14 and CD16 surface markers, three different subpopulations were detected and occurred in the control group in a frequency comparable to the current literature^17^: classical (CD14^++^CD16^−^, 83.3%), intermediate (CD14^++^CD16^+^, 6.1%) and nonclassical (CD14^+^CD16^++^, 9.8%) (Supplementary Table S2). Compared to control samples, we observed significantly enhanced circulating total monocytes in all groups (Fig. 1C) and distinguished the same pattern for the classical (Fig. 1D) and intermediate (Fig. 1E) subsets. A different picture emerged in the nonclassical (Fig. 1F) subpopulation: compared to control samples, we detected significantly decreased levels in the two acute event groups MI and unstable CAD and similar levels in stable CAD patients. A gender-specific subgroup analysis did not reveal major differences between male and female patients. However, we lost some significance levels due to smaller group size (Supplementary Table S2). In addition, we analyzed the flow cytometry data of the monocyte subpopulations regarding the CD14 and CD16 cell surface expression. Compared to control, CD14 expression was upregulated in all patients’ groups in all subsets, whereas CD16 expression was not changed at all (Supplementary Figure S2). Subsequently, we were able to validate the identified subsets by expression profiling of already established subset markers (Supplementary Figure S3) like CCR2 or Nur77. The expression profiles are according to the current literature^4,18^ and prove our gating and RNA isolation strategy.

### Genome-wide transcriptional profiling of monocyte subpopulations from patients with acute MI, stable CAD and control samples

**Figure 2:**
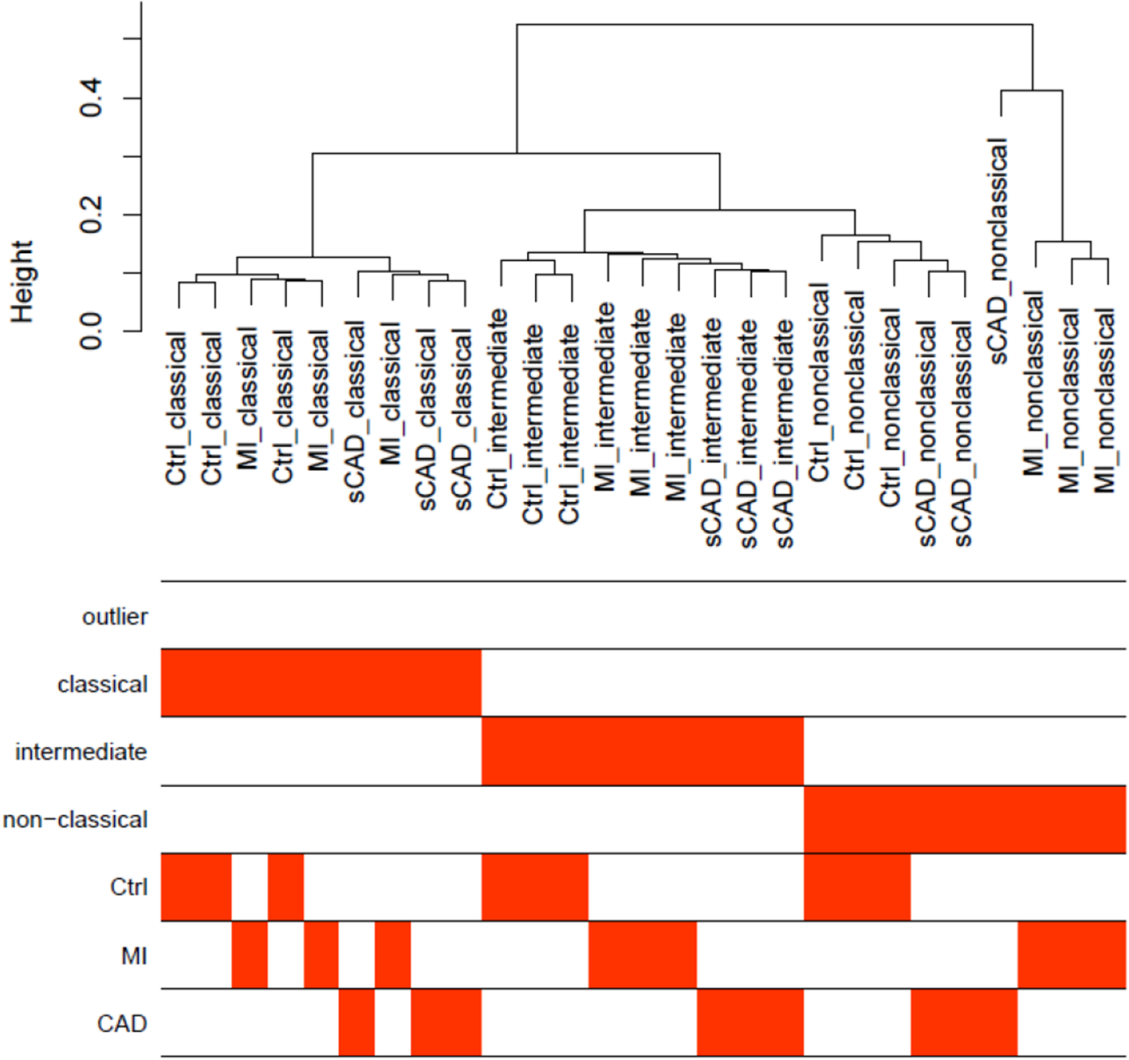
Analysis of miRNA expression raw data from sorted blood monocyte subpopulations of controls, MI patients and CAD patients. Hierarchical clustering of control and patients’ samples are shown as sample dendrogram and trait heat map.

Since we did not see obvious differences in our flow cytometric analysis regarding the distribution of monocytes between patients having a first MI compared to patients having unstable CAD we focused our analysis on acute MI and stable CAD patients. To characterize the expression profiles of these groups, we performed miRNA array and mRNASeq experiments on the three monocyte subpopulations of three male subjects. Interestingly, hierarchical clustering of both data sets revealed that the state of the different monocyte subtypes strongly influences the expression pattern of each subgroup, while the differences of the phenotypes on the patterns appear to be marginal (Fig. 2). Additionally, the profiles of the intermediate and nonclassical monocytes are more related to each other as to the classical monocytes. This finding is in accordance with previous expression array studies^18^. Therefore, we first focused on the characterization of the different monocyte subsets’ expression profiles in the control sample group before elaborating on the phenotype.

### Transcriptional characterization of the three monocyte subsets in control samples

**Figure 3:**
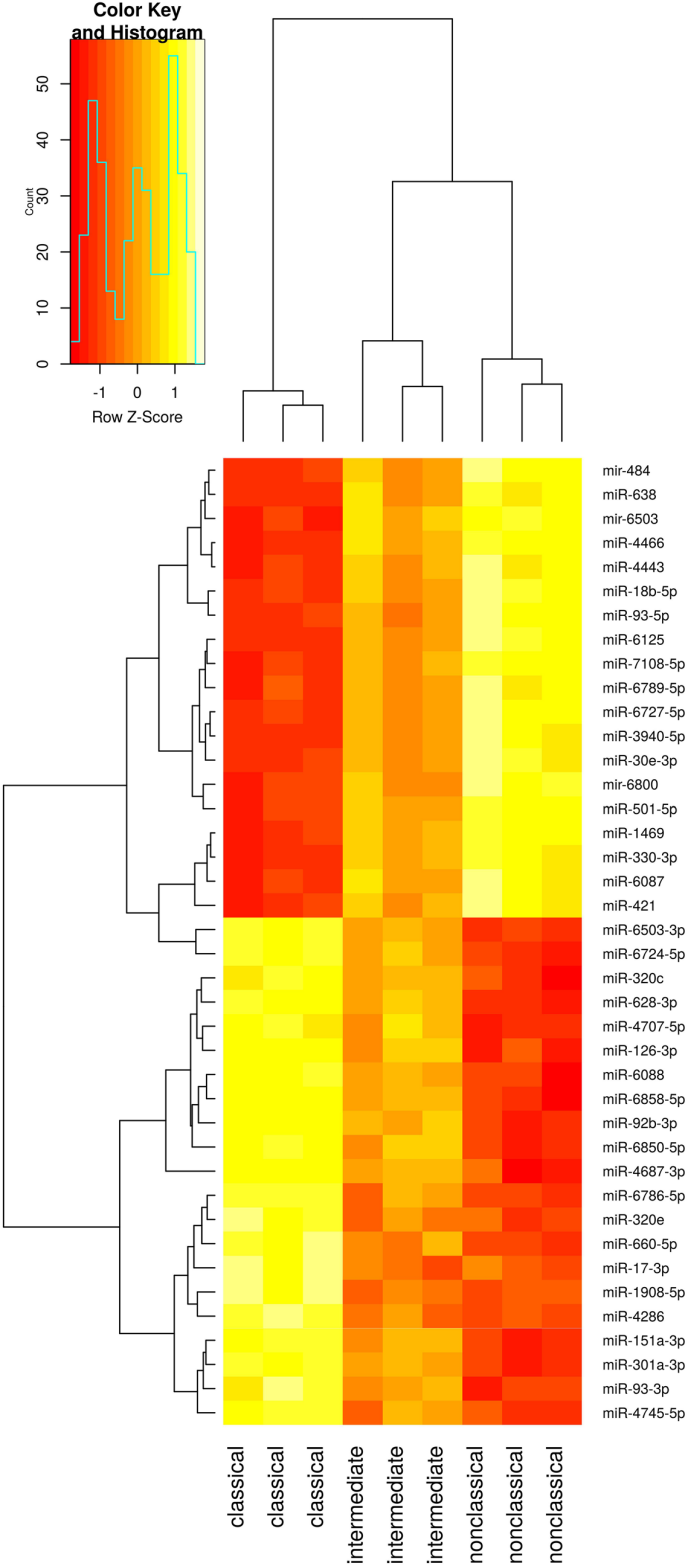
Heat map presentation based on hierarchical clustering of the top significantly expressed miRNAs between classical and nonclassical monocytes in control samples. The values for the particular miRNAs for the intermediate monocyte subset represent the transitional character of this population.

The majority of miRNAs and mRNAs were differentially expressed between the classical and nonclassical subsets, suggesting that indeed the intermediate monocytes represent a transitional population as suggested by previous studies^15,18^. The overall expression of significantly regulated miRNAs and mRNAs exhibits a clear trend of intermediate expression (Supplementary Figure S4). Due to the heterogeneity of our mRNASeq data (see supplementary Table S3) we decided to rely on differentially expressed miRNAs for the next steps. The transitional character of the intermediate monocyte subpopulation is strengthened by a heat map presentation of this data (Fig. 3).

The classical and the nonclassical monocytes show distinct patterns of expression clearly separated from each other while the intermediate subpopulation is in between. In summary we found 320 miRNAs to be differentially expressed (FC >|2|, q ≤ 0.05 (BH adjusted p value), full list is provided in Supplementary Table S4. The most significant miRNAs were miR-151a-3p, miR-6503-3p and miR-126-3p (downregulated in nonclassical monocytes). To further investigate the contrast of transcription patterns between the classical and nonclassical monocyte population and to functionally characterize the differentially expressed miRNAs, we performed correlation analysis between these subsets. We correlated the expression values of the most differentially expressed miRNAs (logFC >  ± 4) and their validated mRNA targets with a particular focus on highly negatively correlated (Pearson’s r ≤ − 0.85, see Table 1). Interestingly, one of the most prominent mRNA targets is the Cyclin-dependent Kinase 6 (CDK6), a well-known regulator of cell differentiation in different cell types as hematopoietic stem cells^19^.

**Table 1:** Top differentially expressed miRNAs (logFC >  ± 4) with negatively correlated mRNA targets (Pearson's correlation r ≤ − 0.85). The logarithmic fold change (FC) is given for the expression between classical and nonclassical monocytes in the control group.

miRNA	Target gene	logFC	q-value
miR-223-3p	ATM	4.59	1.84E−05
miR-484	CDK6; CHORDC1; OGA; USP24; ITPKB; LNPEP; NOL9; SLX4; ZBTB4; ENTPD4; STK10; KIF21B	5.12	4.86E−07
miR-27b-3p	CDK6; SGPP1; RFTN1; KDM3A; ITPKB; HEG1; RALGAPB; AHSA2P; NFAT5; E2F1; ARAP2	4.98	8.13E−08
miR-93-3p	CDK6; TUBGCP6; SLC25A42; ZBTB4; MCM7; MGA; BNC2; KAT6A; GSE1; TRAPPC10; NFATC3; ELP1; NFAT5; SFI1	4.78	8.37E−10
miR-25-3p	CDK6; LNPEP; SCRN1; GUCY1A1; DBT; DUSP5; ITPKB; NFAT5; SEMA4C	5.21	4.86E−07
miR-4429	TSTD2; VPS13D; UBLCP1; UBN2	4.18	1.29E−07
miR-126-3p	PIK3R1; ABLIM1; ZNF331; IL11RA; RASA3; PRR5	4.73	8.37E−10
miR-199a-3p	CDK6; PIK3R1; AVL9; LUC7L; MAP4K5; UHMK1; AKAP11; KMT2D; LNPEP; CHORDC1; SMG1; GIGYF1; AP1G1; CREBZF; MYSM1; MARCHF6; HEG1; RNF213; ARAP2	4.55	1.16E−06
miR-18a-5p	EPHA4; KMT2A; TMEM181; RASA2; ABI2; CREBZF; TRAPPC10; SPATA13; ATM; KDM3A	4.83	5.92E−07
miR-221-3p	CDK6; NKTR; BAZ2A; ARIH2; PIK3R1; ZKSCAN8; DGKE; NFATC3; TRIM33; DYRK2; TRANK1; CREBZF; UBN2; GSE1; HSH2D; SMARCA2; ZBTB37; DDX17; EVL; ARAP2; LNPEP; HEG1	4.21	1.81E−05
miR-199b-3p	AVL9; LUC7L; MAP4K5; AKAP11; CHORDC1; GIGYF1; AP1G1; CREBZF; MYSM1; HEG1	4.55	1.16E−06
miR-486-5p	DYRK1A; CNOT6; UHMK1; PIK3R1	4.09	5.97E−07
miR-18b-5p	CCNL2; MARCHF6; UHMK1; ASCC3; ARAP2	4.55	1.05E−08
miR-145-5p	CDK6; PPM1L; DDX6; ELK4; ARAP2; SPEN; BDP1; SMAD5; BPTF; PRRC2C; AP1G1; ARNTL; USP37; KDM3A; TNRC6B; KDM5A; MARCHF6; BAZ2A; DDX17	4.01	1.49E−05
miR-151a-3p	CDK6; MGA; HEG1	4.38	6.90E−10
miR-320e	CDK6; BAZ2A; C3; OGA; LENG8; DYRK2; JMY; HERC1; NOL4L; LNPEP; AC002316.1; NFAT5	4.22	2.20E−09
miR-421	CDK6; CASP3; INPP4A; ATG2B; CREBZF; DDX17; RASA3; RBBP7; XIAP; RALGAPB; NFAT5; CYFIP2	5.69	8.59E−09
miR-4443	NFAT5; ZBTB37	4.10	1.23E−08
miR-424-3p	CDK6; MARCHF6; MGA; CD47	4.74	1.03E−07
miR-660-5p	CDK6	4.13	1.76E−08

### Transcriptional characterization of the monocyte subsets in patients suffering from acute myocardial infarction and stable coronary artery disease

**Figure 4:**
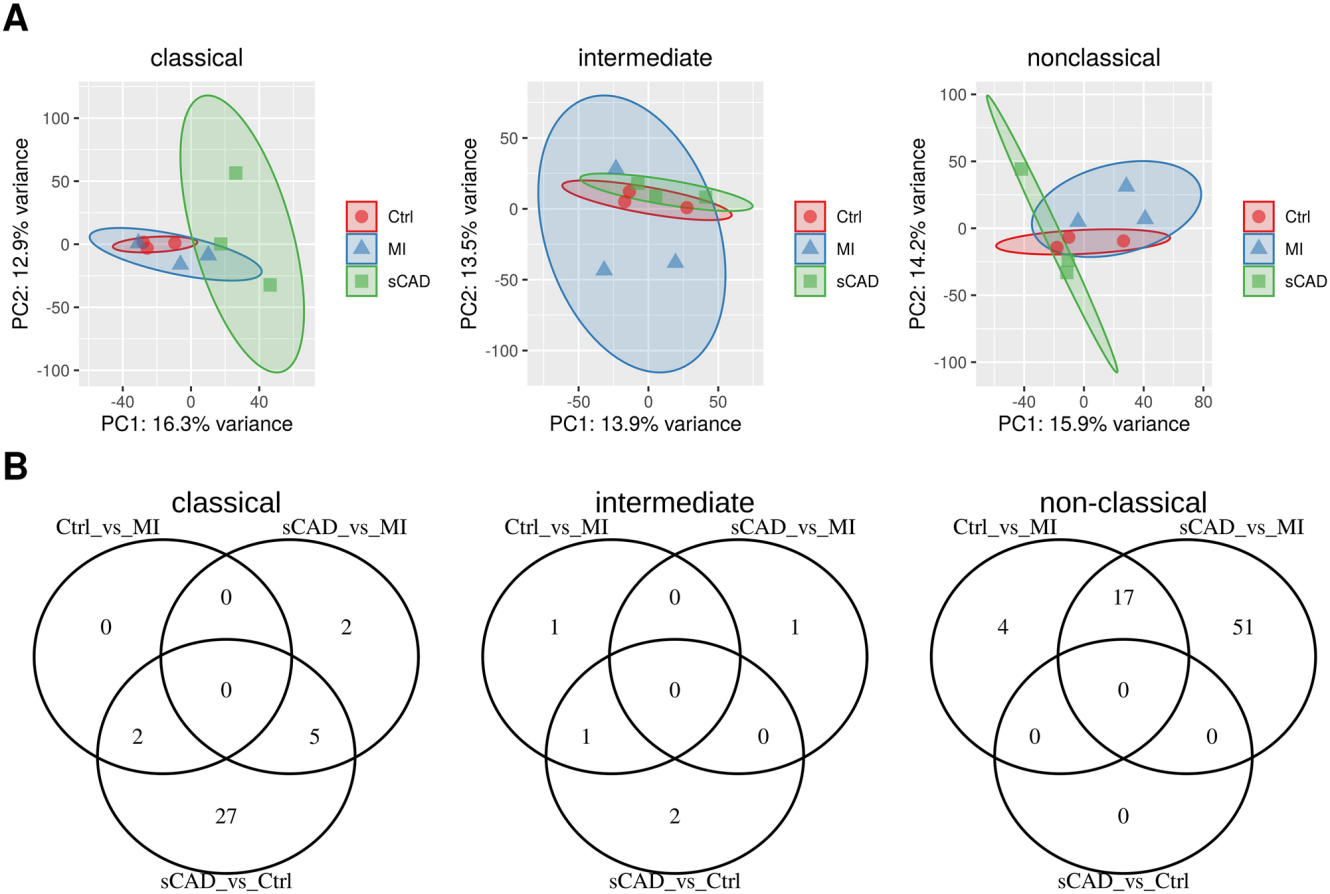
(**A**) Principal component analysis (PCA) plot of the different monocyte subsets for the investigated control samples (Ctrl) and the two diseased phenotypes, patients suffering from an acute myocardial infarction (MI) and stable coronary artery disease (sCAD) based on miRNA expression. (**B**) The majority of differentially expressed miRNAs between phenotypes highlights the importance of the classical monocytes for CAD and nonclassical monocytes for MI.

After establishing the expression profiles in control individuals, we aimed to investigate the differences in expression in the respective disease phenotypes of acute MI and stable CAD. The expression profile of the miRNAs in the classical and nonclassical monocytes distinguishes between the different phenotypes (see Fig. 4A).

Principal component analysis (PCA) of the different subsets shows, that there is a separation of the phenotypes at least in the classical and nonclassical monocytes. In the intermediate subset there is no clear trend, supporting the hypothesis of a transitional population. Our analysis revealed a significant shift in miRNA expression in classical monocytes for patients suffering from CAD and in the nonclassical monocytes for patients with acute MI, which reflects on the number of differentially expressed miRNAs (Fig. 4B). Therefore, we investigated these miRNAs in their respective subsets further for their function by identifying their targets.

### Monocyte subset miRNA expression in acute myocardial infarction

**Figure 5:**
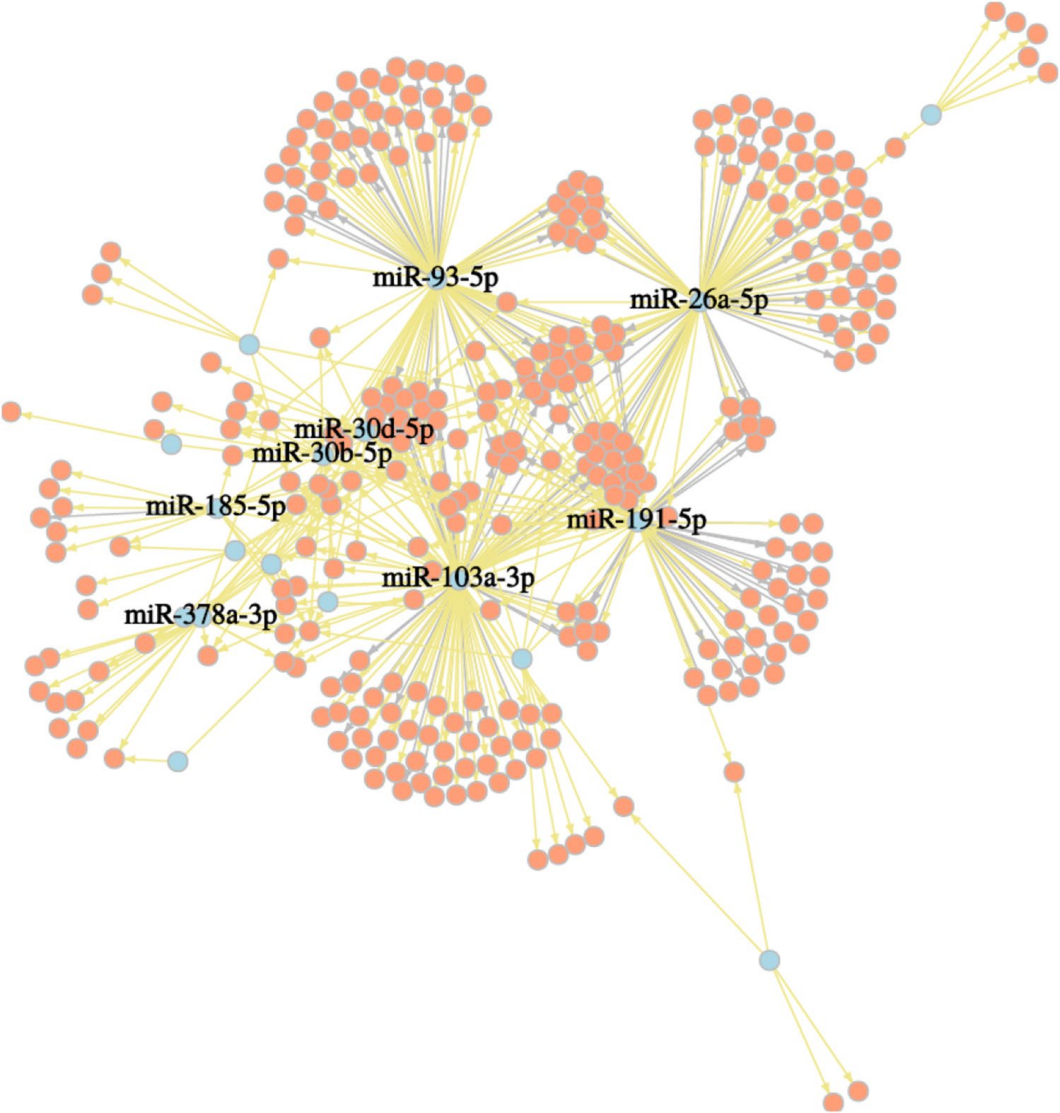
Correlation network of miRNAs that were differentially expressed in MI patients compared to the control samples in the nonclassical monocyte subset. Illustrated are just pairs that correlated more than ± 0.85 (Pearson’s r) and miRNAs having more than 10 targets. Negative correlations are highlighted in yellow, miRNAs in blue and targets in red.

Between control and MI patients, we found 21 (17 specific to MI) miRNAs to be differentially expressed in the nonclassical monocytes, the full list can be found in Supplementary Table S5. The most differentially expressed miRNAs are miR-378(c7a-3p/f/i), miR-422a, miR-26a-5p, miR-532-5p and miR-191-59 (upregulated in our control samples), whereas miR-378 and miR-422a exhibit the same seed sequence and therefore it is assumed that they have similar functions^20^. After correlation analysis, the most strongly correlated miRNA and mRNA target pairs are shown as a correlation network in Fig. 5.

Central miRNAs (all upregulated in our control samples) are miR-191-5p, miR-26a-5p, miR-30d-5p, miR-30b-5p, miR-103a-3p, miR-185-5p, miR-378a-3p and miR-93-5p (associated with angiogenesis by affecting endothelial cell activity^21^). Especially miR-378 has been described in the context of cardiomyocyte hypertrophy and ischemia-induced apoptosis^22,23^. In Table 2 the most significant miRNAs (logFC >  ± 2) for MI with their respective target mRNAs are provided. Targets include apoptosis-related gene XIAP^24^, cell growth and proliferation activator NFAT5^25^ and cardiac cell cycle regulator CDK6^26^. Correlation analysis revealed potential targets like Cyclin D2 (CCND2), B cell lymphoma-2 (BCL2, regulation of apoptosis^27^) and EDARADD. The receptor EDAR and its adapter EDARADD are involved in activation of pro-inflammatory NF-κB pathway^28^. In turn, suppression of NF-κB shows a promising way to prevent ischemia injury^29^. Our data points to the importance of CCND2, as a regulator of CDK kinases, it acts as a cell cycle activator and overexpression is able to enhance myocardial repair^30^. This is in accordance with the established theory of nonclassical monocyte subsets being responsible for patrolling and repair of heart tissue after MI.

**Table 2:** Acute myocardial infarction (MI) specific miRNAs (logFC >  ± 2) and their validated mRNA targets based on correlation analysis (Pearson's correlation r ≤ − 0.85). The logarithmic fold change (FC) is given for the differentially expression in nonclassical monocytes between control and MI patients.

miRNA	mRNA targets	logFC	q-value
miR-185-5p	CCND2; SERPINE2; AC091230.1; HIPK2; ADAMTS1; TUT4; NPC1; EPHA4; SPOCK2; CHD3; MYBL1; ETS1; COL6A2; S1PR1; SLC38A1; ATP8B2	2.06	2.02E−02
miR-378a-3p	CCND2; PLCH2; SPTAN1; LPCAT1; PIM2; ARL4C; NIPA1; NDFIP2; GOLGA8B; STMN1; EDARADD; SYNE2; MDC1; TNRC6C; C1orf21; SPTBN1; GATA3; RHOBTB3; TPX2; TGFBR3; BCL2; ATP2B4; SLC38A1; ITPRIPL1	2.94	1.04E−06
miR-532-5p	CCND2; GDF11; KLRD1; COL6A2; RORA; SYNE1	4.00	3.25E−03
miR-30b-5p	ABCB1; PDGFRB; TRAPPC2; ADAMTS1; CD226; PHLDB2; BCL2; IKZF2; BCL11B; YES1; ZSCAN18; SPATA13; PITPNM2; GOLGA8B; GOLGA8A	2.96	2.02E−02
miR-339-5p	PPP1R16B; ARVCF; ZNF720; SSX2IP	2.83	4.91E−02
miR-378c	MSI2; ARL4C; TNRC6C; RHOBTB3; GATA3; TGFBR3; ITPRIPL1; SLC38A1; BCL2	3.98	5.15E−08
miR-345-5p	CCND2; DMPK; JADE2; STARD9; BCL2; PRSS23; GSE1; F2R; ZBTB37; CDC25B	3.29	5.33E−03
miR-30d-5p	TRAPPC2; SLFN5; LBH; KDM3A; PHLDB2; SPATA13; CD226; TXK; IKZF2; YES1; ADAMTS1; EPHA4; PDGFRB; BCL11B; PITPNM2; GOLGA8B; IFITM1	3.22	6.54E−03
miR-378i	GLCCI1; RORA; TGFBR3; ITPRIPL1; BCL2	3.98	4.64E−04
miR-652-3p	CCND2; ANKRD28; IARS1; BCL9L; ABI2; TRAF5; PLEKHA1	2.92	3.63E−02
miR-422a	SLC38A1; ATP2B4; BCL2	4.03	1.19E−03
miR-324-5p	CCND2; ADGRB2	2.85	1.10E−02
miR-378f	PIM2; BCL2	4.74	1.83E−04

### Monocyte subset miRNA expression in stable coronary artery disease

**Figure 6:**
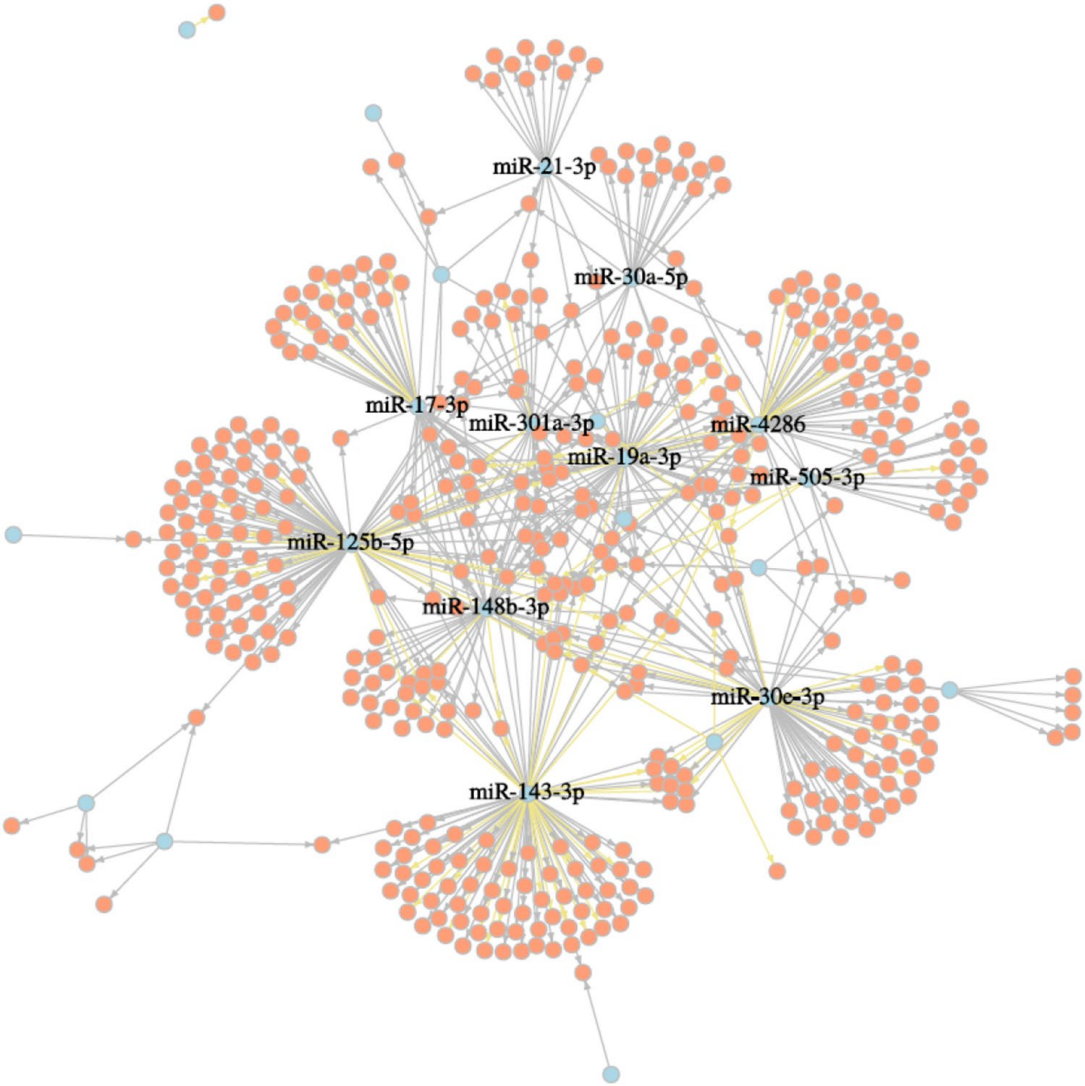
Correlation network of miRNAs that were differentially expressed in CAD patients compared to the controls in the classical monocyte subset. Illustrated are just pairs, that correlated more than ± 0.85 (Pearson’s r) and miRNAs having more than 10 targets. Negative correlations are highlighted in yellow, miRNAs in blue and targets in red.

In total, 34 miRNAs were differentially expressed between patients diagnosed with CAD and control samples in classical monocytes (see Supplementary Table S6 for full list). The five most significant miRNAs are miR-1307-5p, miR-301a-3p, miR-340-5p, miR-30e-3p and miR-148a-3p (upregulated in CAD samples). The correlation network can be seen in Fig. 6. One highly correlated miRNA is miR-17-3p, a member of the mir-17-92 cluster playing a role in cardiomyocyte proliferation^31^. MiR-143-3p levels were already described to be increased in human MI samples^32^.

Of special interest for being further investigated as potential biomarkers are miRNAs that are only differentially expressed in CAD such as miR-301a-3p, miR-30e-3p, miR-194-5p, miR-454-3p and miR-125b-5p (upregulated in CAD samples). In particular, miR-125b (see Fig. 6) was previously shown to play a role in vascular calcification^33^.

## Discussion

Due to their role in adaptive and innate immunity monocytes are of growing interest in inflammation-driven diseases like CAD. In the current study we recruited an extensive, well characterized cohort of patients suffering from different stages of CAD and a set of control patients without any history of cardiovascular disease to evaluate and compare the monocyte subset levels in these stages and to perform, to our best knowledge, a first concurrent flow cytometric and genome-wide miRNA and mRNA profiling approach in all three monocyte subsets. Nevertheless, we must mention common limitations of a study for an age and gender driven disease like CAD: women are underrepresented in a typical study population and a different age between control samples and the diseased patients (Supplemental Table S1). However, we could not detect any correlation between age and monocyte levels in controls, neither in total monocytes, nor in the subpopulations, therefore we can exclude age-dependent effects in this respect (Supplemental Figure S4). In addition, we see no gender-specific effects in our control samples (Supplemental Table S2). Furthermore, we would not assess whether determined changes in circulating subsets reflected the situation in the coronary system. In addition, although we did not include patients with other inflammatory diseases into our study, we could not absolutely rule out the possibility of undetected inflammation. Interestingly, the count pattern between the control and diseased samples are comparable for total, classical and intermediate monocytes. Nonclassical monocytes, in contrast, show a different pattern: the two acute disease phenotypes (MI and unstable CAD) show lower numbers than control or stable CAD samples. This novel observation is explainable by the patrolling and tissue repair function of the subset after an acute event, but it is not consistent to the findings of Tapp et al. reporting no significant changes for this subset after MI^12^.

Emphasizing our second concern, reflecting the expressional changes for the three monocyte subsets in control subjects and patients, we focused on the miRNA profiles: Especially miRNAs as a non-coding RNA species that regulate gene expression in a posttranscriptional manner^34^ and their signature in different cell types and under disease conditions represent interesting targets to investigate in complex regulatory network or functional background. A large number of experimental and clinical studies focused on miRNA expression and function in cardiovascular disease in recent years, including the evaluation of their therapeutic potential^35^. Especially circulating miRNAs as novel biomarkers attracted attention in this regard. We first focused on the differentiation of monocytes from classical to nonclassical subsets in control samples. This part was encouraged by the notable observation that the main transcriptional changes were detected between the three subsets, particularly the classical and nonclassical subset, and not between the different phenotypes. As mentioned above, it is assumed that the intermediate population is a transitory subset between the classical and nonclassical one. All our findings in the miRNA and, in addition, mRNA data support this assumption, and we detected prominent sets of differentially expressed miRNAs and their validated targets that play a role in the differentiation process. Of note, we were able to validate known differentially regulated miRNAs between the two monocyte subsets in control samples, such as miR-19a^36^ or miR-106b^37^, further supporting the notion that distinct miRNAs play a role in the differentiation of these subsets irrespective of disease phenotype. One of the most significantly differentially expressed miRNA marking this process is miR-151a decreasing by 4.38-fold during subset differentiation. miR-151a represents a broadly discussed miRNA biomarker associated with several diseases among others Helicobacter pylori infection and chronic atrophic gastritis^38,39^ or atopic dermatitis^40^ but not in the field of monocyte subsets or differentiation. Interestingly, we were able to detect a high expression correlation to miR-151a target CDK6, a well-known regulator of cell differentiation in different cell types as hematopoietic stem cells^19^. Another differentially expressed miRNA in our data set is miR-223 (FC 4.58, upregulated in classical monocytes in comparison to nonclassical), a well-known player in myeloid differentiation and innate immunity^41^ as the miR-223 level significantly decreases during the differentiation process of monocytes to macrophages. The miRNA was linked to several clinical trials such as rheumatoid arthritis or sepsis in the past^42^. Obviously, further studies should investigate the role of miR-223 as a target in monocyte subset differentiation and the influence of the monocyte subset on different diseases as well. Another study by Zawada et al.^15^ performed smallRNASeq in our control subjects for the three subsets. Concordant with us, they found a major difference in the expression profile of the miRNAs between the classical and nonclassical subsets. Unfortunately, most of the reported miRNAs do not overlap with the results from our experiments, which could be either due to different experimental setup or statistical methods applied. Two miRNAs (miR-20a and miR-106b) are known to being expressed at low levels in nonclassical monocytes after stimulation with LPS. Interestingly, these miRNAs were downregulated in our nonclassical monocyte subsets when analyzing the controls but not in the diseased subjects^43^ in our study suggesting another underlying pathway in response to sepsis.

After carefully characterizing the transcriptional changes in the miRNA profile in the control samples, we set out to mark differences in the disease phenotype of MI and CAD. Surprisingly, we were not able to detect well known miRNAs in the context of inflammation or MI, such as miR-146a^44^. The classical monocytes are generally assumed to accumulate during inflammation, whereas the nonclassical monocytes are patrolling the vasculature and respond to tissue repair^45^. Due to these differences in function, we also hypothesized that this will be reflected in the miRNA expression profiles in the disease phenotypes. In general, we found most involvement of miRNAs specific to MI in the nonclassical monocytes. They confirm the function of nonclassical monocytes during tissue repair, which is an important mechanism after damage of the heart muscle during infarction. On the other hand, the classical subtype of monocytes is most important during inflammatory state of active CAD. These findings highlight the importance of miRNAs during and after disease and can be potentially helpful in prevention, diagnosis and treatment. In general, miRNAs are estimated to be regulating over 60% of the protein-coding genes, which makes them an ideal target in investigating dysregulation in disease phenotypes^46^. Through their stability and abundance in circulating blood, miRNAs pose as especially promising biomarkers, which is why several candidates have been already proposed as such biomarker e.g. miR-208a for myocardial injury^47^, miRNA-548c dilated cardiomypathy^48^ and miR-126 together with members of the miR-17-92 cluster in CAD patients^49^. Additionally monocytes are easily detectable in patients’ blood and can exhibit e.g. uncontrolled inflammatory behavior, which can delay healing after MI^50^, rendering them also great therapeutic targets.

In conclusion we consider our study as a status report to (a) investigate the differentiation of monocytes from classical to nonclassical subsets in our control samples and (b) decipher the role of the different monocyte subsets in MI and CAD. Studies like ours may help to describe specific peculiarities of monocyte subset behavior and cardiovascular disease entities to identify new treatment targets or to predict patients who are likely to respond favorably to treatment. Target analysis of the miRNAs highlights different mechanisms being regulated in the respective phenotypes. In MI patients nonclassical monocytes regulate target genes involved in apoptosis and repair mechanisms, whereas in CAD patients classical monocytes more inflammation and patrolling. Since miRNA have been proven previously to be valuable targets as biomarkers, the miRNAs here pose interesting candidates for further investigation in MI and CAD progression. Especially early diagnosis is in many cases still challenging, and therefore we suggest that these candidates are subject to further investigation of their biomarker or therapeutic potential.

## Methods

### Study population

244 patients undergoing coronary angiography for the diagnosis and percutaneous coronary intervention of CAD were included into this study. Patients received standard cardiovascular care and medication (ACE-inhibitor, AT1-receptor blocker, β -blocker, diuretics, statin and antithrombotic treatment e.g. ASA) according to the actual guidelines. Patients were classified into 3 groups: (1) first event of acute MI (STEMI, NSTEMI n = 110), (2) stable CAD without symptoms within the last 6 months (n = 69) and (3) unstable CAD having a new acute MI (n = 65). In case of MI or unstable CAD blood withdrawal was performed not longer than 4 days after the initial incident. On the basis of standardized questionnaire 61 subjects without any history of cardiovascular disease were included into the study. Exclusion criteria for cases as well as controls were age < 18 or > 70 years, missing of a written informed consent or inability to comply and to understand the investigational nature of the study and the participation at other interventional drug or treatment trials. The study itself was conducted in accordance with the guidelines of the Declaration of Helsinki and the research protocol including the case report forms were approved by the ethics committee of the Medical Faculty, Philipps-University Marburg (#245-12). A written informed consent was obtained from all study participants.

### Blood collection and processing

EDTA blood was collected from each subject, further processing was performed within 2 h. Peripheral blood mononuclear cells (PBMCs) were obtained from 40 mL blood by density gradient centrifugation (Ficoll; Biochrom) at 800*g*, 20 min at room temperature. PBMCs were collected and washed twice at 1500 rpm for 10 min with PBS, the pellet was resuspended in freezing medium Cryo-SFM (Promocell) and aliquots with 5–10 × 10^6^ cells/mL freezing medium per patient or controls were cryopreserved in liquid nitrogen (− 196 °C).

### Flow cytometry, cell sorting and RNA isolation

After washing, PBMCs were stained with anti-human antibodies specific for CD2 (PE, RPA-2.10, T-cell marker), CD14 (APC, M5E2, monocyte subset differentiation), CD15 (PE, HIM1, granulocyte marker), CD16 (PE-Cy7, 3G8, monocyte subset differentiation), CD19 (PE, HIB19, B-cell marker), CD56 (PE, MY31, NK-cell marker), CD335 (PE. 9E2, NK-cell marker), HLA-DR (FITC, TU36, antigen-presenting cells) (all from BD Biosciences) as reported by Cros et al.^16^ DAPI was added to identify dead cells. Cells were acquired on a FACS LSR II flow cytometer (BD Biosciences) and analyzed using FlowJo software version 10 (Treestar Inc.). For sorting of monocyte subsets, PBMCs were treated and stained in 100 μL sorting buffer (PBS, 2 mM EDTA, 2% FCS) with anti-human antibodies as mentioned above. Stained cells were filtered through 70 μm filter and sorted on a MoFlo Astrios cell sorter (Beckman Coulter). Cells were sorted in 1 mL of Isol-RNA lysis reagent (5-Prime GmbH) and frozen at – 80 °C. We selected representative male samples (n = 3) to avoid gender specific effects and subjected them to cell sorting and subsequent RNA isolation. For isolation of total RNA, 200 μL chloroform (Sigma Aldrich) was added per 1 mL Isol-RNA lysis reagent and samples were centrifuged (15,000*g*, 4 °C, 15 min). The RNA containing phase was transferred to 500 μL isopropanol (Sigma Aldrich) for precipitation (2 h, − 20 °C). Glycoblue (Thermo Scientific) was added as a co-precipitant and for pellet visualization. After precipitation, pellet was washed twice with 70% ethanol (10,000*g*, 4 °C, 15 min). The air-dried pellet was resuspended in 12 μL of RNAse free water (5-Prime) and stored at − 80 °C. Based on RNA quality, the samples were forwarded to miRNA array hybridization and NGS library preparation.

### Real-time PCR

Total RNA was reverse transcribed (High Capacity cDNA Reverse Transcription Kit, Thermo Fisher). Real-time (RT) PCR was performed in duplicates using Power SYBR green PCR master mixture on a Step OnePlus RT PCR system (Thermo Fisher). For normalization, expression of β-actin was determined. Relative expression with respect to endogenous control was calculated using the 2–∆CT method (PCR primers see Supplementary Methods).

### Genome-wide miRNA and mRNA profiling

According to the flow cytometry results, 5 representative samples from male study participants from these groups and controls were selected from the biobank stocks and subjected to cell sorting and subsequent RNA isolation. Based on total RNA quality and concentration n = 3 samples for controls, MI patients and CAD patients were selected for transcriptional profiling. Quality control analyses of the total RNA were performed using the Agilent 2100 Small RNA Chip (Agilent Technologies) according to the manufacturers’ instruction. The miRNA abundance of each sample with a total RNA input of 500 ng was evaluated via microarray hybridization. For this purpose, the human Affymetrix GeneChip® miRNA 4.0 Arrays (Affymetrix) were used. The target preparation for GeneChip® miRNA arrays and hybridization were performed using Affymetrix® FlashTag™ Biotin HSR RNA Labeling Kits and GeneChip® Hybridization, Wash, and Stain Kit. The array platform comprised 2578 human mature and 2025 pre-miRNAs from which 55% were found to be significantly expressed among all subpopulations and patient groups. For mRNA profiling using RNA-Seq mRNA of the same samples was enriched using the NEBNext® Poly(A) Magnetic Isolation Module (NEB) followed by cDNA NGS library preparation (NEBNext® Ultra RNA Library Prep Kit for Illumina, NEB). The size of the resulting library was controlled by use of a Bioanalyzer High Sensitivity DNA Kit (Agilent Technologies) und quantified using KAPA Library Quantification Kit for Illumina (Roche). Equimolar pooled libraries were sequenced in a single read mode (75 cycles) on the NextSeq500 System (Illumina) using v2 chemistry yielding in an average QScore distribution of 92% ≥ Q30 score and subsequent demultiplexed and converted to FASTQ files by means of bcl2fastq v2.20 Conversion software (Illumina). 36 M single reads on average per sample were mapped to the reference genome (Supplementary Table S3).

### Expression analysis

The miRNA array raw data was corrected for background noise and normalized (RMA-dabg) using the Affymetrix Expression Console TM software. Differential expression analysis was calculated with the limma package^51^. All miRNAs with a false discovery rate < 0.05 (q-value) and fold-change (FC) > 1.5 were further considered as differentially expressed. Raw data from the poly(A) RNAseq for the same samples was quality controlled using FASTQC^52^ software and trimmed for adapter sequences using Trimmomatic^53^. The resulting reads were mapped to the reference genome GRCh37 by Tophat2^54^, counted by using the R package GenomicAlignments^55^ followed by differential analysis using DEseq2^56^. Due to an extremely low mapping rate, one sample was excluded from further analysis (sCAD nonclassical).

### Investigation of potential miRNA targets

Potential miRNA targets were assessed through the R package multiMiR^57^ with the filter of only validated targets. Further functional investigation of these targets was done through Cytoscape’s ClueGO^58^. Only the most significant GO term enrichments are reported. Correlation analysis between miRNA and mRNA expression was done using the R package rcellminer^59^ and visualized with the R package clusterProfiler^60^.

### Further statistical analysis

Normal distribution of all data was tested using the D’Agostino & Pearson omnibus normality test. Clinical data and monocyte data from flow cytometry were compared using Kruskal–Wallis test followed by Dunn’s multiple comparison test and are presented as box-plot (median with 25th/75th percentile) and whiskers (10th/90th percentile). Data from monocyte marker expression were compared using one-way ANOVA followed by Tukey’s multiple comparison test and are presented as mean with standard deviation of the mean. Spearman’s rank correlation coefficient was used to evaluate possible associations between variables.

## Supplementary Information

Supplementary Information.

## Data Availability

The mRNA Seq datasets generated and analyzed during the current study are available in the NCBI SRA repository, http://www.ncbi.nlm.nih.gov/bioproject/706411. The miRNA array datasets are available in the NCBI GEO repository, https://www.ncbi.nlm.nih.gov/geo/query/acc.cgi?acc=GSE168149.
