# Supplementary material for: Monocyte subpopulation profiling indicates CDK6-derived cell differentiation and identifies subpopulation-specific miRNA expression sets in acute and stable coronary artery disease
Source: Sci Rep. 2022 Apr 4;12:5589. doi: 10.1038/s41598-022-08600-7 (PMC8979987; doi:10.1038/s41598-022-08600-7)
Supplement: Supplementary file 1 — Supplementary Information. [file 41598_2022_8600_MOESM1_ESM.docx]

**Supplemental Data**

1. **Study outline, flow cytometry and cell sorting**

**Supplemental Table S1**

**control MI stable CAD unstable CAD**

n 61 110 69 65

male / female – no. (%) 38/23 (62/38) 93/17 (85/15) 59/10 (86/14) 53/12 (82/18)

age 51.0 (34.2-64.6) 58.0 (50.0-66.0)** 65.0 (51.0-73.0)*** ^###^ 63.0 (50.6-72.4)***

body-mass-index 27.5 (22.5-34.3) 28.1 (23.7-35.6) 29.5 (24.0-38.1) 28.0 (24.1-38.4)

smoking status

current smoker – no. (%) 14 (23) 57 (52) 11 (16) 16 (25)

former smoker – no. (%) 15 (25) 30 (27) 43 (62) 38 (58)

never smoked – no. (%) 32 (52) 23 (21) 15 (22) 11 (17)

blood pressure

systolic (mm Hg) 130.0 (110.0-153.0) 130.0 (110.0-160.0) 130.0 (110.0-164.1) 133.0 (110.0-160.0)

diastolic (mm Hg) 85.0 (70.0-95.0) 80.0 (61.0-90.0)*** 80.0 (60.0-90.0)* 80.0 (68.4-90.0)***

diabetes – no. (%) 5 (8) 18 (16) 25 (36) 18 (28)

hyperlipidemia – no. (%) 13 (21) 99 (90) 62 (90) 61 (94)

hypertension – no. (%) 21 (34) 85 (77) 62 (89) 60 (91)

troponin I (ng/mL) n.d. 1.51 (0.18-11.11)^§§§^ 0.037 (0.02-0.36) 0.630 (0.05-7.16)^§§^

Data are presented as median with 10^th^-90^th^ percentile or n and %, n.d. = not done. *P<0.05, **P<0.01, ***P<0.001 vs. control, ^###^P<0.001 vs. MI, ^§§^P<0.01, ^§§§^P<0.001 vs. stable CAD

**Supplemental Figure S1**

Detailed gating strategy for the identification of human circulating blood monocyte subpopulations based on their relative CD14 and CD16 expression by flow cytometry and cell sorting.

**Supplemental Table S2**

**monocytes control [n=61] MI [n=110] stable CAD [n=69] unstable CAD [n=65]**

**[♂38/♀23] [♂93/♀17] [♂59/♀10] [♂53/♀12]**

total (♂,♀) 60.3 (22.6-82.2) 66.6 (36.6-85.4)* 68.9 (46.7-88.2)** 62.6 (44.6-85.1)*

(♂) 59.8 (23.3-81.0) 67.5 (37.9-85.3)* 69.3 (45.4-86.7)** 62.6 (45.6-85.5)*

(♀) 49.9 (9.3-80.4) 55.6 (16.3-86.1) 68.6 (49.4-90.8)* 65.9 (43.8-85.5)*

classical (♂,♀) 83.3 (59.2-92.4) 86.9 (74.1-92.7)** 85.2 (74.3-93.8)* 87.1 (76.3-94.7)*

(♂) 82.6 (64.8-92.7) 87.1 (74.2-92.9)* 86.2 (75.4-93.7)* 87.1 (76.3-94.9)*

(♀) 84.9 (69.7-91.5) 85.7 (78.1-90.1) 85.6 (74.7-94.0) 88.0 (80.3-93.2)*

intermediate (♂,♀) 6.1 (2.6-13.1) 7.6 (3.5-14.9)** 6.9 (3.7-15.1)* 7.0 (2.8-13.7)*

(♂) 6.5 (2.8-12.4) 7.4 (3.5-14.7)* 6.9 (3.7-13.3) 7.1 (2.9-13.7)*

(♀) 5.7 (2.7-13.2) 7.9 (4.2-14.0)* 6.3 (4.4-15.1) 6.6 (3.9-11.5)*

non-classical (♂,♀) 9.8 (2.9-17.3) 6.0 (2.4-11.7)*^,###^ 8.4 (3.3-19.3) 5.4 (1.3-13.4)**^;##^

(♂) 9.5 (3.3-17.3) 6.1 (2.3-11.3)*^,###^ 8.5 (3.5-18.1) 5.8 (1.3-12.8)^#^

(♀) 8.9 (3.3-12.8) 6.0 (3.0-13.7) 8.0 (4.2-18.7) 5.2 (3.4-12.3)^#^

Data are presented as median with 10^th^−90^th^ percentile.

*P<0.05, **P<0.01, ***P<0.001 vs. control, ^#^P<0.05, ^##^P<0.01, ^###^P<0.001 vs. stable CAD

**Supplemental Figure S2**

CD14 and CD16 expression on monocyte subpopulations from patients with different stages of CAD. Quantification of CD14 and CD16 expression (mean fluorescence intensity, MFI) on classical, intermediate and nonclassical monocyte subpopulations from controls (n=61) and from patients with MI (n=110), stable CAD (n=69) and instable CAD (n=65) by flow cytometry. Data are presented as box plot with median and 25th/75th percentiles (boxes) and 10th/90th percentiles (whiskers).

**Supplemental Figure S3**


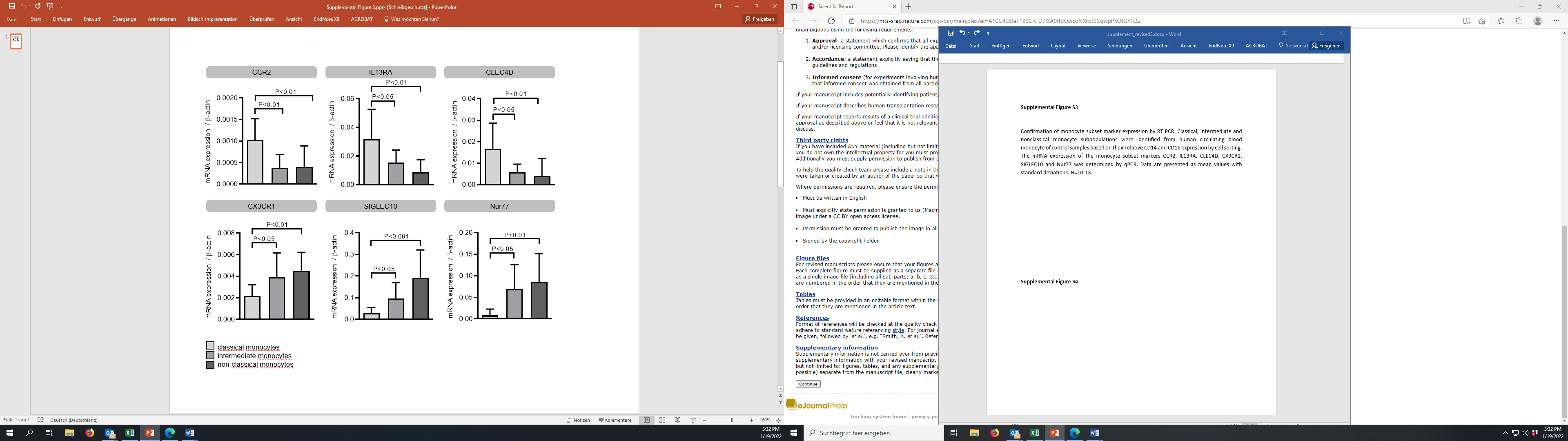


Confirmation of monocyte subset marker expression by RT PCR. Classical, intermediate and nonclassical monocyte subpopulations were identified from human circulating blood monocyte of control samples based on their relative CD14 and CD16 expression by cell sorting. The mRNA expression of the monocyte subset markers CCR2, IL13RA, CLEC4D, CX3CR1, SIGLEC10 and Nur77 was determined by qPCR. Data are presented as mean values with standard deviations. N=10-13.

**Supplemental Figure S4**

Correlation analysis of age and monocyte levels based on their relative CD14 and CD16 expression by flow cytometry in total monocytes, classical monocytes, intermediate monocytes and nonclassical monocytes in control samples (n=61).

1. **Supplemental Methods**

**PCR primer**

PCR primers were obtained from Microsynth (Balgach, Switzerland). Primer sequences are as follows: CCR2 forward: 5´-GGG GAG AAG TTC AGA AGC CTT T-3´, CCR2 reverse: 5´-GGA GTG GGG CAA TCC TAC AG-3´, CLEC4D forward: 5´-CAA TGG GGC TAG AAA AAC CTC-´3, CLEC4D reverse: 5´-GAA GGT ATC AGC TGG GGA TG-3´, CX3CR1 forward: 5´-AAC CCC TGG AGG CGT TTA AG-3´, CX3CR1 reverse: 5´- GAT CCA TGG TGA AGG CCC CA-3´, IL13RA1 forward: 5´-TTC CCT CCA ATT CCT GAT CC-3´, IL13RA1 reverse: 5´-TCC TCC TTG GTT TGC TTC TC-3´, NUR77 forward: 5´-GTT CTC TGG AGG TCA TCC GCA AG-3´, NUR77 reverse: 5´-GCA GGG ACC TTG AGA AGG CCA-3´, SIGLEC10 forward: 5´-TTC ATG AAC GAT GGG TTC TTT-3´, SIGLEC10 reverse: 5´-TCC TCA AAG GCC CAG TTA AA-3´, β-actin forward: 5’-CAT GTA CGT TGC TAT CCA GGC-3’, β-actin reverse: 5’-CTC CTT AAT GTC ACG CAC GAT-3’.

1. **Transcriptional Profiling data**

**Supplemental Table S3:** mRNA Seq statistics

| Sample name | SampleID | Sample type | Number of reads | Mapped reads [%] | Monocyte subset |
| --- | --- | --- | --- | --- | --- |
| BN0078_1 | 1 | Ctrl | 36589704 | 90.5 | classical |
| BN0078_2 | 2 | Ctrl | 36097842 | 84.4 | intermediate |
| BN0078_3 | 3 | Ctrl | 36275330 | 38.2 | nonclassical |
| BN0079_1 | 4 | Ctrl | 36143156 | 59.0 | classical |
| BN0079_2 | 5 | Ctrl | 35719754 | 76.4 | intermediate |
| BN0079_3 | 6 | Ctrl | 36206812 | 49.4 | nonclassical |
| BN0090_1 | 7 | Ctrl | 35984874 | 91.0 | classical |
| BN0090_2 | 8 | Ctrl | 35788430 | 85.7 | intermediate |
| BN0090_3 | 9 | Ctrl | 35646789 | 70.7 | nonclassical |
| BN0165_1 | 10 | MI | 35895364 | 91.0 | classical |
| BN0165_2 | 11 | MI | 35638916 | 86.2 | intermediate |
| BN0165_3 | 12 | MI | 35863524 | 57.2 | nonclassical |
| BN0199_1 | 13 | MI | 35803799 | 91.1 | classical |
| BN0199_2 | 14 | MI | 35619972 | 87.5 | intermediate |
| BN0199_3 | 15 | MI | 36237303 | 49.5 | nonclassical |
| BN0374_1 | 16 | MI | 35793776 | 91.4 | classical |
| BN0374_2 | 17 | MI | 35952621 | 89.4 | intermediate |
| BN0374_3 | 18 | MI | 36279749 | 87.5 | nonclassical |
| BN0015_1 | 19 | sCAD | 35601487 | 90.4 | classical |
| BN0015_2 | 20 | sCAD | 35899277 | 62.8 | intermediate |
| BN0015_3 | 21 | sCAD | 34976367 | 40.2 | nonclassical |
| BN0011_1 | 22 | sCAD | 35885855 | 88.3 | classical |
| BN0011_2 | 23 | sCAD | 35107904 | 76.6 | intermediate |
| BN0011_3 | 24 | sCAD | 36420715 | 5.1 | nonclassical |
| BN0408_1 | 25 | sCAD | 36843591 | 91.7 | classical |
| BN0408_2 | 26 | sCAD | 35403822 | 49.7 | intermediate |
| BN0408_3 | 27 | sCAD | 36295425 | 36.9 | nonclassical |

**Supplemental Figure S4**

Overall expression of the differentially regulated miRNAs and mRNAs between the classical and nonclassical MCs shown for all three subpopulations.


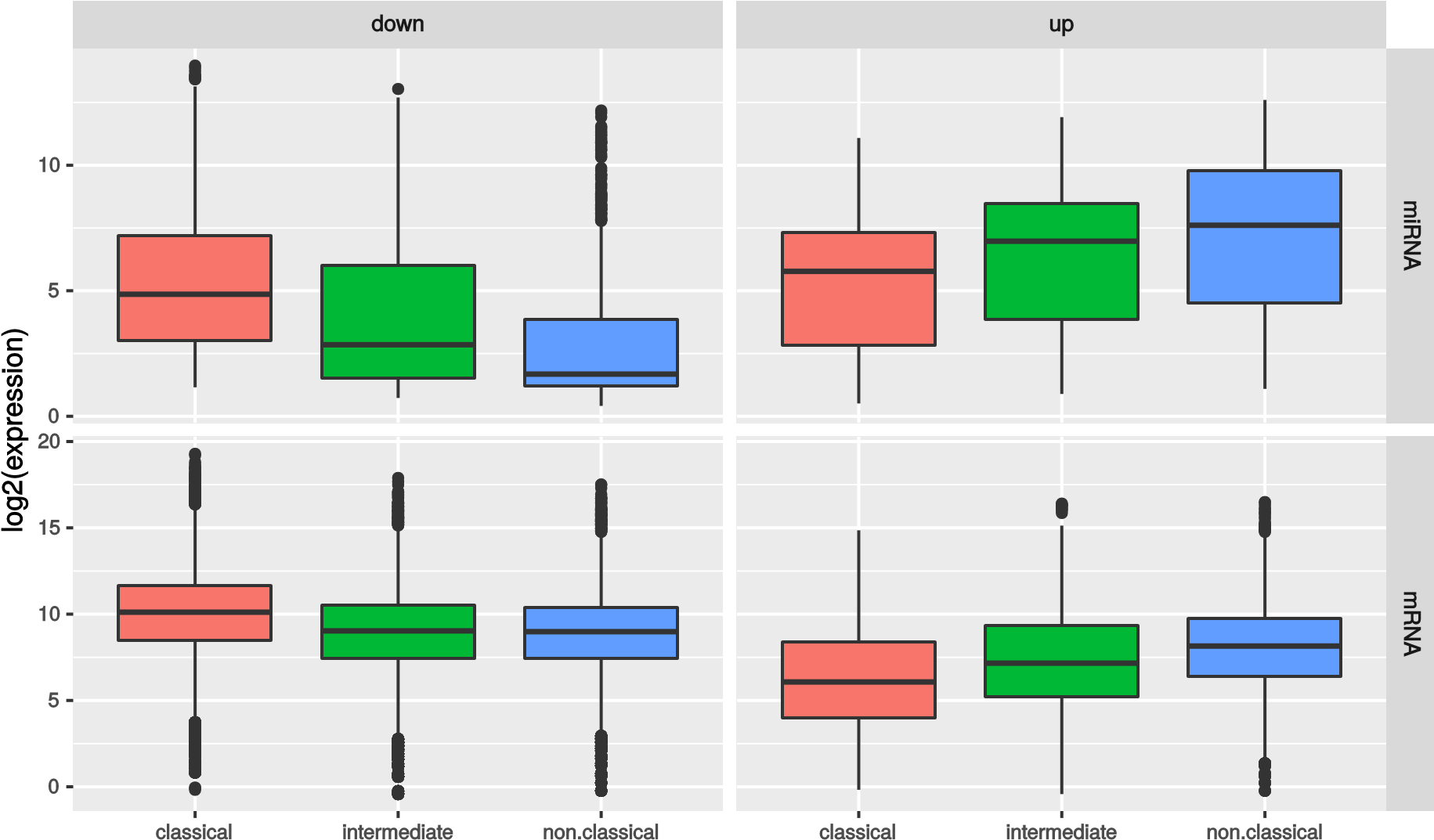


**Supplemental Table S4** Lists of diff. regulated miRNAs (n=320) between classical and nonclassical MCs in control samples. B is the log-odds of the gene being differentially expressed. Moderated t-statistic (t) is the ratio of the M-value to its standard error.

| gene | logFC | CI.L | CI.R | AveExpr | t | P.Value | adj.P.Val | B |
| --- | --- | --- | --- | --- | --- | --- | --- | --- |
| hsa-miR-151a-3p | 4.377 | 4.016 | 4.738 | 3.701 | 15.722 | 1.56E-13 | 6.90E-10 | 21.043 |
| hsa-miR-6503-3p | 4.898 | 4.270 | 5.527 | 3.587 | 15.106 | 3.53E-13 | 7.81E-10 | 20.258 |
| hsa-miR-126-3p | 4.732 | 4.329 | 5.135 | 4.450 | 14.500 | 8.10E-13 | 8.37E-10 | 19.455 |
| hsa-miR-4707-5p | -3.020 | -3.342 | -2.698 | 7.324 | -14.322 | 1.04E-12 | 8.37E-10 | 19. |
| hsa-miR-93-3p | 4.785 | 3.705 | 5.865 | 4.659 | 14.294 | 1.08E-12 | 8.37E-10 | 19.174 |
| hsa-miR-4286 | 3.716 | 3.362 | 4.070 | 2.813 | 14.259 | 1.14E-12 | 8.37E-10 | 19.127 |
| hsa-miR-320e | 4.220 | 3.903 | 4.537 | 4.041 | 13.482 | 3.49E-12 | 2.20E-09 | 18.033 |
| hsa-miR-6858-5p | -3.088 | -4.029 | -2.148 | 7.660 | -13.311 | 4.50E-12 | 2.49E-09 | 17.784 |
| hsa-miR-4745-5p | -2.943 | -3.336 | -2.550 | 7.451 | -13.162 | 5.63E-12 | 2.53E-09 | 17.566 |
| hsa-miR-301a-3p | 3.880 | 3.286 | 4.473 | 2.648 | 13.152 | 5.71E-12 | 2.53E-09 | 17.550 |
| hsa-miR-6786-5p | -2.429 | -2.990 | -1.868 | 9.195 | -13.003 | 7.15E-12 | 2.64E-09 | 17.331 |
| hsa-miR-17-3p | 3.711 | 2.153 | 5.269 | 2.689 | 12.936 | 7.93E-12 | 2.64E-09 | 17.229 |
| hsa-miR-4466 | -2.647 | -3.958 | -1.336 | 10.159 | -12.929 | 8.01E-12 | 2.64E-09 | 17.219 |
| hsa-miR-320c | 2.500 | 1.236 | 3.764 | 9.411 | 12.879 | 8.64E-12 | 2.64E-09 | 17.145 |
| hsa-miR-6850-5p | -3.261 | -3.872 | -2.650 | 7.906 | -12.857 | 8.95E-12 | 2.64E-09 | 17.111 |
| hsa-miR-6724-5p | -2.484 | -2.794 | -2.173 | 8.738 | -12.760 | 1.04E-11 | 2.75E-09 | 16.965 |
| hsa-miR-638 | -2.644 | -2.968 | -2.321 | 9.740 | -12.748 | 1.06E-11 | 2.75E-09 | 16.946 |
| hsa-mir-6800 | -2.345 | -2.676 | -2.015 | 7.648 | -12.529 | 1.49E-11 | 3.65E-09 | 16.612 |
| hsa-miR-6088 | -2.280 | -2.619 | -1.941 | 10.082 | -12.356 | 1.95E-11 | 4.54E-09 | 16.344 |
| hsa-miR-1908-5p | -2.981 | -3.266 | -2.695 | 8.299 | -12.143 | 2.74E-11 | 5.85E-09 | 16.011 |
| hsa-miR-93-5p | 2.928 | 2.597 | 3.258 | 9.831 | 12.133 | 2.78E-11 | 5.85E-09 | 15.996 |
| hsa-miR-4687-3p | -2.343 | -2.728 | -1.958 | 9.187 | -12.103 | 2.92E-11 | 5.86E-09 | 15.948 |
| hsa-miR-1469 | -2.932 | -3.585 | -2.279 | 9.185 | -11.814 | 4.66E-11 | 8.59E-09 | 15.485 |
| hsa-mir-6503 | 2.445 | 2.185 | 2.705 | 2.260 | 11.758 | 5.10E-11 | 8.59E-09 | 15.395 |
| hsa-miR-3940-5p | -2.723 | -3.039 | -2.406 | 8.491 | -11.745 | 5.22E-11 | 8.59E-09 | 15.374 |
| hsa-miR-6727-5p | -2.396 | -3.879 | -0.913 | 9.684 | -11.731 | 5.34E-11 | 8.59E-09 | 15.350 |
| hsa-miR-421 | 5.686 | 5.409 | 5.963 | 4.055 | 11.727 | 5.37E-11 | 8.59E-09 | 15.345 |
| hsa-miR-330-3p | 2.952 | 1.900 | 4.004 | 2.670 | 11.720 | 5.44E-11 | 8.59E-09 | 15.333 |
| hsa-miR-6087 | -1.717 | -2.079 | -1.356 | 10.883 | -11.679 | 5.81E-11 | 8.86E-09 | 15.267 |
| hsa-miR-92b-3p | 3.238 | 2.963 | 3.513 | 2.864 | 11.594 | 6.70E-11 | 9.87E-09 | 15.127 |
| hsa-miR-18b-5p | 4.554 | 3.844 | 5.265 | 3.000 | 11.536 | 7.37E-11 | 1.05E-08 | 15.031 |
| hsa-miR-4443 | 4.100 | 3.704 | 4.496 | 6.035 | 11.418 | 8.97E-11 | 1.23E-08 | 14.838 |
| hsa-mir-484 | 3.900 | 3.606 | 4.194 | 4.315 | 11.383 | 9.51E-11 | 1.23E-08 | 14.780 |
| hsa-miR-501-5p | 3.543 | 3.303 | 3.782 | 3.157 | 11.379 | 9.57E-11 | 1.23E-08 | 14.773 |
| hsa-miR-6125 | -1.963 | -2.370 | -1.556 | 10.251 | -11.368 | 9.76E-11 | 1.23E-08 | 14.754 |
| hsa-miR-7108-5p | -2.478 | -2.795 | -2.160 | 7.845 | -11.344 | 1.02E-10 | 1.25E-08 | 14.715 |
| hsa-miR-6789-5p | -3.999 | -4.357 | -3.642 | 8.035 | -11.224 | 1.24E-10 | 1.49E-08 | 14.513 |
| hsa-miR-628-3p | 2.851 | 2.512 | 3.190 | 2.207 | 11.097 | 1.55E-10 | 1.76E-08 | 14.298 |
| hsa-miR-660-5p | 4.131 | 3.798 | 4.465 | 4.069 | 11.095 | 1.55E-10 | 1.76E-08 | 14.295 |
| hsa-miR-30e-3p | 3.850 | 2.747 | 4.953 | 2.252 | 11.027 | 1.74E-10 | 1.92E-08 | 14.180 |
| hsa-miR-4787-5p | -1.809 | -2.647 | -0.971 | 10.641 | -11.011 | 1.79E-10 | 1.93E-08 | 14.152 |
| hsa-miR-27a-5p | 3.712 | 3.344 | 4.079 | 3.021 | 10.976 | 1.90E-10 | 2.00E-08 | 14.092 |
| hsa-miR-652-5p | 2.520 | 2.117 | 2.923 | 2.208 | 10.943 | 2.01E-10 | 2.07E-08 | 14.037 |
| hsa-miR-8069 | -2.502 | -4.075 | -0.928 | 10.205 | -10.818 | 2.50E-10 | 2.51E-08 | 13.821 |
| hsa-miR-8072 | -2.717 | -3.467 | -1.966 | 8.402 | -10.645 | 3.38E-10 | 3.32E-08 | 13.520 |
| hsa-miR-2861 | -2.288 | -2.533 | -2.043 | 8.932 | -10.610 | 3.60E-10 | 3.46E-08 | 13.459 |
| hsa-miR-320a | 2.634 | 2.244 | 3.023 | 9.592 | 10.548 | 4.02E-10 | 3.78E-08 | 13.348 |
| hsa-miR-331-3p | 3.465 | 3.023 | 3.907 | 3.009 | 10.529 | 4.16E-10 | 3.82E-08 | 13.315 |
| hsa-miR-3196 | -2.768 | -3.084 | -2.452 | 9.436 | -10.519 | 4.23E-10 | 3.82E-08 | 13.298 |
| hsa-miR-4488 | -3.042 | -3.718 | -2.365 | 9.379 | -10.244 | 6.92E-10 | 6.12E-08 | 12.808 |
| hsa-miR-6089 | -2.014 | -2.544 | -1.484 | 11.414 | -10.210 | 7.36E-10 | 6.38E-08 | 12.746 |
| hsa-miR-1237-5p | -2.535 | -2.861 | -2.209 | 9.131 | -10.091 | 9.13E-10 | 7.77E-08 | 12.531 |
| hsa-miR-27b-3p | 4.985 | 4.482 | 5.487 | 4.391 | 10.056 | 9.74E-10 | 8.13E-08 | 12.467 |
| hsa-miR-1915-3p | -2.298 | -3.146 | -1.450 | 9.849 | -10.037 | 1.01E-09 | 8.26E-08 | 12.432 |
| hsa-miR-149-3p | -2.062 | -2.421 | -1.703 | 8.726 | -10.022 | 1.04E-09 | 8.33E-08 | 12.405 |
| hsa-miR-320b | 2.478 | 1.812 | 3.144 | 9.478 | 10.008 | 1.06E-09 | 8.40E-08 | 12.379 |
| hsa-miR-424-3p | 4.740 | 4.230 | 5.251 | 3.958 | 9.888 | 1.33E-09 | 1.03E-07 | 12.158 |
| hsa-miR-4429 | 4.180 | 3.744 | 4.615 | 5.408 | 9.758 | 1.69E-09 | 1.29E-07 | 11.918 |
| hsa-miR-6821-5p | -2.045 | -2.387 | -1.703 | 7.652 | -9.748 | 1.72E-09 | 1.29E-07 | 11.900 |
| hsa-miR-4734 | -2.429 | -2.856 | -2.003 | 7.834 | -9.738 | 1.75E-09 | 1.29E-07 | 11.880 |
| hsa-miR-128-3p | 3.952 | 3.275 | 4.628 | 2.924 | 9.660 | 2.03E-09 | 1.47E-07 | 11.734 |
| hsa-miR-6765-5p | -2.402 | -3.436 | -1.368 | 6.671 | -9.630 | 2.15E-09 | 1.53E-07 | 11.678 |
| hsa-miR-6729-5p | -1.946 | -2.329 | -1.563 | 10.182 | -9.615 | 2.21E-09 | 1.55E-07 | 11.651 |
| hsa-miR-328-3p | 3.583 | 3.196 | 3.970 | 3.800 | 9.590 | 2.31E-09 | 1.60E-07 | 11.604 |
| hsa-miR-3621 | -3.783 | -4.144 | -3.422 | 6.878 | -9.547 | 2.51E-09 | 1.71E-07 | 11.522 |
| hsa-miR-6090 | -1.878 | -2.813 | -0.943 | 11.406 | -9.519 | 2.65E-09 | 1.77E-07 | 11.468 |
| hsa-miR-148b-3p | 3.910 | 3.541 | 4.280 | 2.721 | 9.437 | 3.09E-09 | 2.04E-07 | 11.313 |
| hsa-miR-4516 | -2.280 | -2.836 | -1.723 | 10.130 | -9.383 | 3.43E-09 | 2.23E-07 | 11.210 |
| hsa-miR-125b-5p | 2.937 | 2.606 | 3.268 | 2.149 | 9.316 | 3.90E-09 | 2.48E-07 | 11.082 |
| hsa-miR-320d | 2.970 | 2.657 | 3.283 | 7.458 | 9.313 | 3.92E-09 | 2.48E-07 | 11.075 |
| hsa-miR-4674 | -3.540 | -3.874 | -3.207 | 7.838 | -9.290 | 4.10E-09 | 2.55E-07 | 11.032 |
| hsa-miR-6816-5p | -2.800 | -3.210 | -2.389 | 7.765 | -9.239 | 4.52E-09 | 2.78E-07 | 10.934 |
| hsa-miR-1228-5p | -2.423 | -2.738 | -2.108 | 7.459 | -9.232 | 4.58E-09 | 2.78E-07 | 10.920 |
| hsa-miR-20a-5p | 3.567 | 3.207 | 3.927 | 9.168 | 9.223 | 4.67E-09 | 2.79E-07 | 10.902 |
| hsa-miR-941 | 4.053 | 2.792 | 5.314 | 3.628 | 9.214 | 4.75E-09 | 2.80E-07 | 10.885 |
| hsa-miR-425-3p | 4.332 | 3.957 | 4.708 | 4.632 | 9.199 | 4.88E-09 | 2.84E-07 | 10.857 |
| hsa-miR-6800-5p | -2.520 | -2.838 | -2.201 | 8.218 | -9.188 | 4.99E-09 | 2.86E-07 | 10.836 |
| hsa-miR-4281 | -1.899 | -2.446 | -1.351 | 6.957 | -9.139 | 5.49E-09 | 3.11E-07 | 10.740 |
| hsa-miR-3656 | -2.347 | -2.548 | -2.146 | 8.485 | -9.044 | 6.61E-09 | 3.70E-07 | 10.554 |
| hsa-miR-4270 | -2.765 | -3.136 | -2.394 | 7.635 | -8.989 | 7.36E-09 | 4.07E-07 | 10.446 |
| hsa-miR-18a-3p | 3.217 | 2.364 | 4.070 | 2.517 | 8.972 | 7.60E-09 | 4.15E-07 | 10.414 |
| hsa-miR-484 | 5.119 | 4.786 | 5.451 | 4.198 | 8.880 | 9.12E-09 | 4.86E-07 | 10.232 |
| hsa-miR-25-3p | 5.209 | 4.066 | 6.352 | 6.361 | 8.873 | 9.24E-09 | 4.86E-07 | 10.218 |
| hsa-miR-191-5p | 1.861 | 1.493 | 2.228 | 11.970 | 8.873 | 9.24E-09 | 4.86E-07 | 10.218 |
| hsa-miR-18a-5p | 4.834 | 3.694 | 5.975 | 5.977 | 8.768 | 1.14E-08 | 5.92E-07 | 10.009 |
| hsa-miR-486-5p | 4.088 | 3.650 | 4.525 | 3.243 | 8.758 | 1.16E-08 | 5.97E-07 | 9.989 |
| hsa-miR-1273h-3p | 2.570 | 1.028 | 4.111 | 2.350 | 8.683 | 1.35E-08 | 6.85E-07 | 9.840 |
| hsa-miR-550a-3p | 2.965 | 2.416 | 3.514 | 2.258 | 8.668 | 1.39E-08 | 6.99E-07 | 9.809 |
| hsa-miR-663a | -3.328 | -3.605 | -3.052 | 7.765 | -8.596 | 1.61E-08 | 7.98E-07 | 9.665 |
| hsa-miR-762 | -1.782 | -3.074 | -0.490 | 9.318 | -8.581 | 1.66E-08 | 8.14E-07 | 9.633 |
| hsa-miR-500a-5p | 3.415 | 1.663 | 5.166 | 5.069 | 8.571 | 1.69E-08 | 8.15E-07 | 9.614 |
| hsa-miR-3665 | -1.864 | -2.163 | -1.565 | 11.400 | -8.569 | 1.70E-08 | 8.15E-07 | 9.610 |
| hsa-miR-362-5p | 3.945 | 3.432 | 4.459 | 5.147 | 8.435 | 2.22E-08 | 1.06E-06 | 9.339 |
| hsa-miR-486-3p | 3.121 | 2.876 | 3.367 | 2.607 | 8.430 | 2.25E-08 | 1.06E-06 | 9.329 |
| hsa-miR-1307-5p | 2.425 | -0.313 | 5.164 | 1.642 | 8.375 | 2.51E-08 | 1.16E-06 | 9.215 |
| hsa-miR-106b-3p | 4.174 | 3.418 | 4.930 | 5.640 | 8.368 | 2.55E-08 | 1.16E-06 | 9.201 |
| hsa-miR-199a-3p | 4.550 | 4.030 | 5.069 | 3.287 | 8.363 | 2.58E-08 | 1.16E-06 | 9.191 |
| hsa-miR-199b-3p | 4.550 | 3.849 | 5.250 | 3.287 | 8.363 | 2.58E-08 | 1.16E-06 | 9.191 |
| hsa-miR-143-3p | 2.957 | 2.264 | 3.651 | 2.212 | 8.346 | 2.67E-08 | 1.19E-06 | 9.155 |
| hsa-miR-6803-5p | -1.604 | -1.936 | -1.273 | 8.527 | -8.280 | 3.06E-08 | 1.35E-06 | 9.020 |
| hsa-miR-328-5p | -2.263 | -3.600 | -0.926 | 7.158 | -8.093 | 4.49E-08 | 1.97E-06 | 8.633 |
| hsa-miR-5787 | -1.713 | -2.389 | -1.037 | 9.385 | -8.065 | 4.76E-08 | 2.06E-06 | 8.575 |
| hsa-miR-501-3p | 3.445 | 2.359 | 4.532 | 4.978 | 8.021 | 5.23E-08 | 2.24E-06 | 8.481 |
| hsa-miR-25-5p | 2.521 | 1.926 | 3.115 | 2.439 | 7.945 | 6.12E-08 | 2.60E-06 | 8.323 |
| hsa-miR-532-3p | 3.699 | 3.141 | 4.257 | 5.538 | 7.940 | 6.19E-08 | 2.61E-06 | 8.312 |
| hsa-miR-6732-5p | -2.706 | -2.995 | -2.418 | 8.429 | -7.918 | 6.49E-08 | 2.71E-06 | 8.264 |
| hsa-miR-378g | 3.101 | 2.666 | 3.535 | 2.824 | 7.853 | 7.43E-08 | 3.07E-06 | 8.129 |
| hsa-miR-6805-5p | -2.508 | -2.945 | -2.071 | 6.816 | -7.798 | 8.35E-08 | 3.42E-06 | 8.011 |
| hsa-let-7e-5p | 3.201 | 2.631 | 3.772 | 3.498 | 7.781 | 8.66E-08 | 3.51E-06 | 7.975 |
| hsa-miR-326 | 3.176 | 2.463 | 3.888 | 2.013 | 7.684 | 1.07E-07 | 4.28E-06 | 7.767 |
| hsa-miR-194-5p | 2.469 | 2.167 | 2.770 | 2.567 | 7.611 | 1.24E-07 | 4.96E-06 | 7.611 |
| hsa-miR-17-5p | 3.038 | 2.751 | 3.325 | 10.095 | 7.604 | 1.26E-07 | 4.99E-06 | 7.596 |
| hsa-miR-6791-5p | -1.711 | -2.014 | -1.408 | 7.690 | -7.535 | 1.46E-07 | 5.73E-06 | 7.447 |
| hsa-miR-148a-3p | 2.750 | 2.437 | 3.062 | 1.615 | 7.510 | 1.55E-07 | 6.00E-06 | 7.393 |
| hsa-miR-3178 | -2.927 | -3.259 | -2.595 | 6.550 | -7.487 | 1.62E-07 | 6.21E-06 | 7.343 |
| hsa-miR-188-5p | 2.443 | 2.057 | 2.829 | 2.192 | 7.486 | 1.63E-07 | 6.21E-06 | 7.340 |
| hsa-miR-1227-5p | -2.337 | -2.993 | -1.682 | 5.922 | -7.474 | 1.67E-07 | 6.31E-06 | 7.315 |
| hsa-miR-23a-5p | 3.461 | 2.461 | 4.460 | 4.042 | 7.471 | 1.68E-07 | 6.31E-06 | 7.308 |
| hsa-miR-199b-5p | 1.909 | 1.488 | 2.331 | 1.512 | 7.426 | 1.85E-07 | 6.89E-06 | 7.211 |
| hsa-miR-6752-5p | -1.518 | -2.873 | -0.163 | 6.359 | -7.416 | 1.89E-07 | 6.98E-06 | 7.190 |
| hsa-miR-454-3p | 1.973 | 0.762 | 3.184 | 1.604 | 7.377 | 2.06E-07 | 7.53E-06 | 7.104 |
| hsa-miR-21-3p | 2.730 | 1.106 | 4.355 | 2.149 | 7.369 | 2.10E-07 | 7.60E-06 | 7.087 |
| hsa-mir-4417 | -1.786 | -2.188 | -1.384 | 3.864 | -7.354 | 2.17E-07 | 7.80E-06 | 7.052 |
| hsa-miR-19a-3p | 3.258 | 2.930 | 3.587 | 2.626 | 7.346 | 2.21E-07 | 7.87E-06 | 7.036 |
| hsa-miR-629-5p | 3.695 | 3.069 | 4.321 | 3.727 | 7.323 | 2.32E-07 | 8.21E-06 | 6.985 |
| hsa-mir-185 | 1.145 | -0.080 | 2.371 | 1.441 | 7.314 | 2.37E-07 | 8.30E-06 | 6.966 |
| hsa-miR-29b-2-5p | 2.659 | 1.699 | 3.619 | 2.531 | 7.290 | 2.49E-07 | 8.68E-06 | 6.914 |
| hsa-miR-505-5p | 3.180 | 2.830 | 3.529 | 3.375 | 7.272 | 2.60E-07 | 8.97E-06 | 6.872 |
| hsa-miR-625-5p | 3.660 | 3.223 | 4.097 | 3.132 | 7.231 | 2.84E-07 | 9.73E-06 | 6.783 |
| hsa-miR-4497 | -2.125 | -2.505 | -1.745 | 8.529 | -7.207 | 2.99E-07 | 1.02E-05 | 6.731 |
| hsa-miR-7704 | -1.683 | -2.091 | -1.275 | 10.510 | -7.198 | 3.05E-07 | 1.03E-05 | 6.711 |
| hsa-miR-1185-2-3p | 2.324 | 1.855 | 2.793 | 1.869 | 7.193 | 3.09E-07 | 1.03E-05 | 6.699 |
| hsa-miR-130b-3p | 3.777 | 3.488 | 4.067 | 5.908 | 7.131 | 3.54E-07 | 1.17E-05 | 6.562 |
| hsa-miR-4763-3p | -1.707 | -2.228 | -1.186 | 7.116 | -7.128 | 3.56E-07 | 1.17E-05 | 6.557 |
| hsa-miR-3615 | 4.729 | 4.309 | 5.150 | 4.021 | 7.095 | 3.83E-07 | 1.25E-05 | 6.482 |
| hsa-miR-1185-1-3p | 1.967 | 1.641 | 2.292 | 1.918 | 7.090 | 3.87E-07 | 1.26E-05 | 6.472 |
| hsa-miR-145-5p | 4.011 | 3.727 | 4.295 | 3.232 | 7.011 | 4.61E-07 | 1.49E-05 | 6.295 |
| hsa-miR-26a-5p | 1.746 | 1.003 | 2.490 | 11.562 | 6.964 | 5.12E-07 | 1.64E-05 | 6.191 |
| hsa-miR-4485 | 4.511 | 4.195 | 4.828 | 3.780 | 6.913 | 5.74E-07 | 1.81E-05 | 6.075 |
| hsa-miR-221-3p | 4.215 | 2.976 | 5.454 | 7.447 | 6.911 | 5.77E-07 | 1.81E-05 | 6.071 |
| hsa-miR-4284 | 2.651 | 1.891 | 3.411 | 3.586 | 6.910 | 5.77E-07 | 1.81E-05 | 6.070 |
| hsa-miR-130a-3p | 3.560 | 2.706 | 4.413 | 3.122 | 6.898 | 5.93E-07 | 1.84E-05 | 6.043 |
| hsa-miR-103a-3p | 2.124 | 1.780 | 2.467 | 12.174 | 6.894 | 5.99E-07 | 1.84E-05 | 6.032 |
| hsa-miR-223-3p | 4.587 | 3.457 | 5.716 | 6.248 | 6.893 | 6.01E-07 | 1.84E-05 | 6.031 |
| hsa-miR-3651 | 3.074 | 1.888 | 4.260 | 2.760 | 6.855 | 6.53E-07 | 1.99E-05 | 5.946 |
| hsa-miR-181d-5p | 2.519 | 1.861 | 3.176 | 2.218 | 6.852 | 6.58E-07 | 1.99E-05 | 5.939 |
| hsa-miR-151a-5p | 3.808 | 2.680 | 4.935 | 6.046 | 6.840 | 6.76E-07 | 2.03E-05 | 5.912 |
| hsa-miR-671-3p | 2.471 | 2.100 | 2.842 | 2.246 | 6.838 | 6.79E-07 | 2.03E-05 | 5.908 |
| hsa-miR-340-5p | 1.031 | 0.690 | 1.372 | 1.362 | 6.790 | 7.56E-07 | 2.24E-05 | 5.799 |
| hsa-miR-550a-5p | 1.501 | 1.185 | 1.816 | 1.723 | 6.748 | 8.32E-07 | 2.45E-05 | 5.704 |
| hsa-miR-24-2-5p | 3.464 | 2.636 | 4.292 | 3.201 | 6.734 | 8.58E-07 | 2.51E-05 | 5.672 |
| hsa-miR-500b-3p | 2.376 | 2.058 | 2.695 | 1.832 | 6.726 | 8.75E-07 | 2.54E-05 | 5.653 |
| hsa-mir-93 | 1.449 | 1.099 | 1.798 | 1.681 | 6.697 | 9.33E-07 | 2.69E-05 | 5.588 |
| hsa-miR-30a-5p | 2.300 | 1.103 | 3.498 | 1.977 | 6.696 | 9.36E-07 | 2.69E-05 | 5.585 |
| hsa-miR-151b | 3.182 | 2.841 | 3.523 | 2.438 | 6.687 | 9.54E-07 | 2.72E-05 | 5.566 |
| hsa-miR-6718-5p | 2.580 | 2.220 | 2.940 | 1.738 | 6.671 | 9.90E-07 | 2.81E-05 | 5.529 |
| hsa-miR-378a-5p | 3.264 | 2.837 | 3.690 | 4.317 | 6.663 | 1.01E-06 | 2.84E-05 | 5.511 |
| hsa-miR-4739 | -1.854 | -3.683 | -0.024 | 7.692 | -6.649 | 1.04E-06 | 2.90E-05 | 5.479 |
| hsa-miR-5001-5p | -1.647 | -2.279 | -1.015 | 7.198 | -6.648 | 1.04E-06 | 2.90E-05 | 5.476 |
| hsa-miR-3960 | -1.162 | -1.586 | -0.738 | 11.618 | -6.642 | 1.06E-06 | 2.92E-05 | 5.463 |
| hsa-miR-324-3p | 3.586 | 3.315 | 3.856 | 3.475 | 6.636 | 1.07E-06 | 2.94E-05 | 5.449 |
| hsa-miR-20b-5p | 3.803 | 3.403 | 4.203 | 6.462 | 6.578 | 1.22E-06 | 3.34E-05 | 5.318 |
| hsa-miR-339-3p | 2.682 | 2.347 | 3.017 | 4.257 | 6.568 | 1.25E-06 | 3.37E-05 | 5.295 |
| hsa-miR-505-3p | 2.281 | 1.951 | 2.612 | 1.852 | 6.568 | 1.25E-06 | 3.37E-05 | 5.295 |
| hsa-miR-21-5p | 3.420 | 3.124 | 3.716 | 4.181 | 6.561 | 1.27E-06 | 3.40E-05 | 5.279 |
| hsa-miR-378e | 2.679 | 2.330 | 3.028 | 2.056 | 6.529 | 1.37E-06 | 3.65E-05 | 5.204 |
| hsa-miR-1273h-5p | 1.181 | 0.083 | 2.279 | 1.900 | 6.504 | 1.45E-06 | 3.83E-05 | 5.148 |
| hsa-miR-199a-5p | 2.218 | 1.001 | 3.435 | 1.724 | 6.451 | 1.64E-06 | 4.31E-05 | 5.025 |
| hsa-miR-6503-5p | 2.995 | 2.323 | 3.667 | 2.078 | 6.442 | 1.67E-06 | 4.37E-05 | 5.004 |
| hsa-mir-6722 | -1.760 | -2.055 | -1.466 | 6.259 | -6.423 | 1.74E-06 | 4.54E-05 | 4.961 |
| hsa-miR-1207-5p | -1.275 | -3.045 | 0.495 | 6.500 | -6.308 | 2.27E-06 | 5.87E-05 | 4.696 |
| hsa-miR-542-5p | 1.741 | 1.424 | 2.059 | 1.528 | 6.290 | 2.37E-06 | 6.10E-05 | 4.653 |
| hsa-miR-491-5p | 2.597 | 1.972 | 3.221 | 2.165 | 6.281 | 2.42E-06 | 6.18E-05 | 4.633 |
| hsa-miR-494-3p | 3.097 | 2.634 | 3.560 | 2.520 | 6.273 | 2.46E-06 | 6.26E-05 | 4.614 |
| hsa-miR-574-3p | 4.325 | 3.853 | 4.796 | 6.488 | 6.270 | 2.48E-06 | 6.26E-05 | 4.608 |
| hsa-miR-1973 | 2.076 | 1.805 | 2.346 | 1.870 | 6.241 | 2.66E-06 | 6.67E-05 | 4.539 |
| hsa-miR-4306 | 2.781 | 2.144 | 3.417 | 2.253 | 6.219 | 2.80E-06 | 6.99E-05 | 4.487 |
| hsa-miR-185-3p | 1.894 | 1.158 | 2.630 | 1.751 | 6.209 | 2.86E-06 | 7.08E-05 | 4.465 |
| hsa-miR-92a-3p | 2.556 | 1.754 | 3.358 | 10.853 | 6.208 | 2.87E-06 | 7.08E-05 | 4.463 |
| hsa-miR-103a-2-5p | 1.767 | -0.227 | 3.761 | 1.654 | 6.169 | 3.14E-06 | 7.71E-05 | 4.371 |
| hsa-miR-2110 | 2.848 | 1.779 | 3.918 | 3.271 | 6.159 | 3.21E-06 | 7.85E-05 | 4.348 |
| hsa-miR-671-5p | 2.168 | 1.910 | 2.426 | 3.119 | 6.124 | 3.49E-06 | 8.48E-05 | 4.266 |
| hsa-miR-425-5p | 2.293 | 1.972 | 2.614 | 9.417 | 6.117 | 3.54E-06 | 8.56E-05 | 4.251 |
| hsa-miR-193a-5p | 2.745 | 1.841 | 3.650 | 5.427 | 6.087 | 3.80E-06 | 9.12E-05 | 4.181 |
| hsa-miR-30b-3p | 1.581 | 1.342 | 1.820 | 1.811 | 6.041 | 4.23E-06 | 1.01E-04 | 4.072 |
| hsa-mir-92b | 1.716 | 1.322 | 2.110 | 1.582 | 6.040 | 4.25E-06 | 1.01E-04 | 4.069 |
| hsa-miR-181a-3p | 3.109 | 1.186 | 5.032 | 3.463 | 6.037 | 4.27E-06 | 1.01E-04 | 4.063 |
| hsa-mir-320e | -1.802 | -2.143 | -1.461 | 7.975 | -6.006 | 4.60E-06 | 1.08E-04 | 3.989 |
| hsa-miR-200c-3p | 3.446 | 3.177 | 3.715 | 4.318 | 5.979 | 4.89E-06 | 1.14E-04 | 3.927 |
| hsa-miR-99a-5p | 2.113 | 1.345 | 2.880 | 1.879 | 5.952 | 5.22E-06 | 1.21E-04 | 3.862 |
| hsa-miR-3175 | 2.588 | 2.242 | 2.933 | 1.902 | 5.903 | 5.85E-06 | 1.35E-04 | 3.748 |
| hsa-miR-345-5p | 3.290 | 2.131 | 4.450 | 6.173 | 5.874 | 6.26E-06 | 1.44E-04 | 3.679 |
| hsa-miR-152-3p | 3.158 | 2.827 | 3.488 | 3.547 | 5.845 | 6.70E-06 | 1.54E-04 | 3.611 |
| hsa-miR-107 | 1.968 | 0.902 | 3.034 | 11.709 | 5.824 | 7.06E-06 | 1.61E-04 | 3.560 |
| hsa-mir-21 | 1.025 | 0.638 | 1.412 | 1.549 | 5.792 | 7.60E-06 | 1.72E-04 | 3.486 |
| hsa-miR-1233-5p | -2.864 | -4.651 | -1.077 | 5.814 | -5.791 | 7.63E-06 | 1.72E-04 | 3.481 |
| hsa-let-7b-5p | 1.595 | 0.088 | 3.102 | 11.168 | 5.790 | 7.64E-06 | 1.72E-04 | 3.480 |
| hsa-miR-500a-3p | 3.285 | 1.673 | 4.896 | 5.566 | 5.783 | 7.77E-06 | 1.73E-04 | 3.464 |
| hsa-miR-502-3p | 3.579 | 3.191 | 3.967 | 5.608 | 5.694 | 9.61E-06 | 2.12E-04 | 3.251 |
| hsa-miR-106a-5p | 3.010 | 2.073 | 3.947 | 9.780 | 5.658 | 1.05E-05 | 2.30E-04 | 3.166 |
| hsa-miR-28-3p | 3.353 | 3.026 | 3.680 | 5.641 | 5.609 | 1.18E-05 | 2.57E-04 | 3.049 |
| hsa-miR-155-5p | 3.937 | 3.094 | 4.779 | 7.083 | 5.501 | 1.52E-05 | 3.30E-04 | 2.791 |
| hsa-miR-1260a | 1.611 | 1.068 | 2.153 | 1.856 | 5.474 | 1.62E-05 | 3.50E-04 | 2.727 |
| hsa-miR-769-5p | 1.945 | 0.566 | 3.324 | 2.162 | 5.456 | 1.70E-05 | 3.64E-04 | 2.682 |
| hsa-miR-7977 | 3.035 | 2.767 | 3.302 | 4.673 | 5.441 | 1.76E-05 | 3.75E-04 | 2.648 |
| hsa-miR-100-5p | 2.369 | 1.559 | 3.179 | 1.845 | 5.425 | 1.83E-05 | 3.88E-04 | 2.610 |
| hsa-miR-142-5p | 1.982 | 1.482 | 2.483 | 1.735 | 5.407 | 1.91E-05 | 4.04E-04 | 2.565 |
| hsa-miR-363-3p | 3.190 | 2.353 | 4.026 | 2.809 | 5.394 | 1.97E-05 | 4.15E-04 | 2.533 |
| hsa-miR-423-5p | 2.535 | 2.119 | 2.951 | 6.524 | 5.324 | 2.33E-05 | 4.88E-04 | 2.366 |
| hsa-miR-192-5p | 2.147 | 1.489 | 2.804 | 1.687 | 5.320 | 2.35E-05 | 4.90E-04 | 2.357 |
| hsa-mir-423 | 1.711 | 1.319 | 2.104 | 4.812 | 5.317 | 2.37E-05 | 4.92E-04 | 2.350 |
| hsa-mir-106b | 1.091 | 0.570 | 1.611 | 1.100 | 5.300 | 2.47E-05 | 5.10E-04 | 2.308 |
| hsa-miR-378d | 2.470 | 2.015 | 2.924 | 4.081 | 5.265 | 2.69E-05 | 5.50E-04 | 2.224 |
| hsa-miR-4741 | -2.027 | -2.576 | -1.477 | 5.678 | -5.248 | 2.80E-05 | 5.71E-04 | 2.183 |
| hsa-miR-4467 | -1.818 | -2.170 | -1.465 | 5.740 | -5.190 | 3.22E-05 | 6.54E-04 | 2.042 |
| hsa-miR-589-3p | 2.322 | 2.024 | 2.620 | 2.116 | 5.177 | 3.32E-05 | 6.71E-04 | 2.012 |
| hsa-mir-500a | 1.224 | 0.897 | 1.550 | 1.652 | 5.159 | 3.47E-05 | 6.97E-04 | 1.969 |
| hsa-miR-19b-3p | 2.979 | 1.427 | 4.531 | 7.196 | 5.155 | 3.51E-05 | 7.03E-04 | 1.957 |
| hsa-mir-885 | -1.258 | -1.588 | -0.928 | 2.395 | -5.142 | 3.62E-05 | 7.21E-04 | 1.927 |
| hsa-miR-6775-5p | -1.290 | -2.002 | -0.578 | 7.194 | -5.109 | 3.92E-05 | 7.74E-04 | 1.847 |
| hsa-miR-503-5p | 3.313 | 3.010 | 3.615 | 3.374 | 5.064 | 4.38E-05 | 8.61E-04 | 1.737 |
| hsa-miR-106b-5p | 3.284 | 2.887 | 3.680 | 8.668 | 5.022 | 4.85E-05 | 9.45E-04 | 1.636 |
| hsa-let-7c-5p | 1.765 | 1.420 | 2.110 | 9.499 | 4.989 | 5.25E-05 | 1.02E-03 | 1.556 |
| hsa-miR-1343-5p | -2.162 | -2.626 | -1.697 | 5.339 | -4.984 | 5.32E-05 | 1.03E-03 | 1.544 |
| hsa-miR-140-5p | 3.092 | 2.801 | 3.382 | 4.188 | 4.887 | 6.73E-05 | 1.29E-03 | 1.309 |
| hsa-miR-3147 | -1.647 | -2.854 | -0.441 | 2.120 | -4.855 | 7.29E-05 | 1.39E-03 | 1.230 |
| hsa-let-7i-5p | 2.952 | 2.586 | 3.319 | 8.327 | 4.853 | 7.31E-05 | 1.39E-03 | 1.227 |
| hsa-miR-22-3p | 1.267 | 0.713 | 1.820 | 9.229 | 4.822 | 7.90E-05 | 1.49E-03 | 1.150 |
| hsa-miR-7975 | 2.107 | 1.384 | 2.831 | 2.464 | 4.796 | 8.40E-05 | 1.58E-03 | 1.089 |
| hsa-miR-933 | 1.992 | 1.561 | 2.423 | 1.920 | 4.727 | 9.96E-05 | 1.87E-03 | 0.920 |
| hsa-miR-1270 | 1.514 | 1.216 | 1.813 | 1.410 | 4.699 | 1.07E-04 | 1.99E-03 | 0.852 |
| hsa-miR-5100 | 2.826 | 2.365 | 3.286 | 6.376 | 4.647 | 1.21E-04 | 2.24E-03 | 0.725 |
| hsa-miR-1246 | 2.253 | 1.225 | 3.280 | 1.926 | 4.635 | 1.25E-04 | 2.30E-03 | 0.695 |
| hsa-miR-1273g-3p | 1.397 | 1.123 | 1.671 | 9.284 | 4.591 | 1.39E-04 | 2.55E-03 | 0.590 |
| hsa-miR-99b-5p | 2.075 | 1.437 | 2.714 | 2.173 | 4.572 | 1.46E-04 | 2.66E-03 | 0.544 |
| hsa-mir-6776 | -1.892 | -2.244 | -1.541 | 4.601 | -4.553 | 1.53E-04 | 2.78E-03 | 0.496 |
| hsa-miR-29b-1-5p | 1.828 | 1.284 | 2.372 | 1.801 | 4.497 | 1.75E-04 | 3.16E-03 | 0.360 |
| hsa-miR-8075 | -1.807 | -2.776 | -0.837 | 7.666 | -4.485 | 1.81E-04 | 3.24E-03 | 0.331 |
| hsa-miR-1301-3p | 2.247 | 1.945 | 2.549 | 4.218 | 4.479 | 1.83E-04 | 3.27E-03 | 0.318 |
| hsa-miR-1260b | 2.847 | 2.551 | 3.143 | 4.349 | 4.472 | 1.86E-04 | 3.30E-03 | 0.302 |
| hsa-miR-629-3p | 1.819 | 0.751 | 2.886 | 2.431 | 4.463 | 1.90E-04 | 3.36E-03 | 0.279 |
| hsa-miR-7641 | 3.775 | 3.270 | 4.279 | 5.549 | 4.434 | 2.04E-04 | 3.60E-03 | 0.208 |
| hsa-miR-6861-5p | 1.597 | 1.198 | 1.995 | 1.788 | 4.414 | 2.15E-04 | 3.75E-03 | 0.159 |
| hsa-miR-766-3p | 1.895 | 1.531 | 2.259 | 4.546 | 4.378 | 2.35E-04 | 4.08E-03 | 0.071 |
| hsa-miR-6790-5p | -2.159 | -2.578 | -1.740 | 4.012 | -4.351 | 2.50E-04 | 4.33E-03 | 0.007 |
| hsa-miR-4706 | -1.627 | -1.911 | -1.342 | 3.523 | -4.349 | 2.52E-04 | 4.33E-03 | 0.002 |
| hsa-mir-941-1 | 1.229 | 0.954 | 1.505 | 2.583 | 4.324 | 2.68E-04 | 4.53E-03 | -0.058 |
| hsa-mir-941-2 | 1.229 | 0.915 | 1.544 | 2.583 | 4.324 | 2.68E-04 | 4.53E-03 | -0.058 |
| hsa-mir-941-3 | 1.229 | 0.932 | 1.526 | 2.583 | 4.324 | 2.68E-04 | 4.53E-03 | -0.058 |
| hsa-mir-941-4 | 1.229 | 0.812 | 1.646 | 2.583 | 4.324 | 2.68E-04 | 4.53E-03 | -0.058 |
| hsa-miR-6771-5p | -2.242 | -2.818 | -1.666 | 5.928 | -4.319 | 2.71E-04 | 4.56E-03 | -0.070 |
| hsa-let-7g-5p | 2.692 | 1.980 | 3.404 | 6.998 | 4.319 | 2.71E-04 | 4.56E-03 | -0.072 |
| hsa-miR-30e-5p | 3.205 | 2.901 | 3.509 | 4.153 | 4.309 | 2.78E-04 | 4.65E-03 | -0.095 |
| hsa-miR-4486 | -2.602 | -3.006 | -2.198 | 3.407 | -4.294 | 2.88E-04 | 4.81E-03 | -0.131 |
| hsa-miR-338-5p | 1.515 | 0.756 | 2.274 | 1.648 | 4.292 | 2.90E-04 | 4.82E-03 | -0.137 |
| hsa-miR-371b-5p | 1.934 | 1.596 | 2.272 | 2.319 | 4.289 | 2.92E-04 | 4.83E-03 | -0.143 |
| hsa-miR-24-3p | 1.937 | 1.612 | 2.262 | 11.151 | 4.282 | 2.97E-04 | 4.89E-03 | -0.160 |
| hsa-miR-1275 | 2.042 | 1.698 | 2.386 | 3.948 | 4.279 | 2.99E-04 | 4.92E-03 | -0.168 |
| hsa-miR-3185 | -2.092 | -2.472 | -1.713 | 5.146 | -4.204 | 3.60E-04 | 5.87E-03 | -0.349 |
| hsa-miR-324-5p | 2.195 | 1.916 | 2.474 | 4.738 | 4.189 | 3.73E-04 | 6.06E-03 | -0.385 |
| hsa-miR-660-3p | 1.269 | 0.986 | 1.552 | 1.332 | 4.170 | 3.91E-04 | 6.31E-03 | -0.432 |
| hsa-miR-222-3p | 2.430 | 2.077 | 2.783 | 8.422 | 4.160 | 4.00E-04 | 6.43E-03 | -0.455 |
| hsa-miR-652-3p | 2.548 | 2.223 | 2.873 | 7.243 | 4.158 | 4.03E-04 | 6.45E-03 | -0.461 |
| hsa-mir-361 | 2.083 | 1.789 | 2.378 | 6.322 | 4.106 | 4.57E-04 | 7.27E-03 | -0.585 |
| hsa-miR-6743-5p | -1.191 | -1.531 | -0.850 | 6.944 | -4.098 | 4.66E-04 | 7.36E-03 | -0.605 |
| hsa-miR-221-5p | 1.308 | -0.200 | 2.816 | 1.488 | 4.069 | 5.01E-04 | 7.89E-03 | -0.676 |
| hsa-miR-15b-5p | 3.497 | 3.182 | 3.811 | 8.338 | 4.056 | 5.17E-04 | 8.08E-03 | -0.707 |
| hsa-mir-5703 | -1.003 | -1.347 | -0.659 | 3.336 | -4.049 | 5.26E-04 | 8.20E-03 | -0.724 |
| hsa-miR-6722-3p | -1.499 | -1.860 | -1.137 | 5.848 | -4.040 | 5.37E-04 | 8.30E-03 | -0.744 |
| hsa-miR-378c | 1.196 | -0.115 | 2.507 | 7.408 | 4.030 | 5.51E-04 | 8.46E-03 | -0.769 |
| hsa-miR-6836-5p | 1.632 | 1.324 | 1.939 | 1.987 | 3.979 | 6.25E-04 | 9.49E-03 | -0.892 |
| hsa-miR-619-5p | 2.427 | 2.117 | 2.737 | 3.190 | 3.952 | 6.66E-04 | 1.00E-02 | -0.955 |
| hsa-mir-5095 | -1.324 | -1.692 | -0.956 | 4.093 | -3.952 | 6.66E-04 | 1.00E-02 | -0.955 |
| hsa-mir-3714 | 1.038 | 0.589 | 1.486 | 1.436 | 3.925 | 7.13E-04 | 1.07E-02 | -1.021 |
| hsa-miR-550a-3-5p | 1.208 | 0.865 | 1.551 | 1.574 | 3.915 | 7.29E-04 | 1.09E-02 | -1.044 |
| hsa-mir-550a-1 | -1.434 | -1.693 | -1.175 | 3.967 | -3.903 | 7.51E-04 | 1.10E-02 | -1.073 |
| hsa-mir-550a-2 | -1.434 | -1.765 | -1.102 | 3.967 | -3.903 | 7.51E-04 | 1.10E-02 | -1.073 |
| hsa-mir-550a-3 | -1.434 | -1.781 | -1.087 | 3.967 | -3.903 | 7.51E-04 | 1.10E-02 | -1.073 |
| hsa-miR-3180-3p | -2.305 | -2.636 | -1.975 | 3.556 | -3.858 | 8.39E-04 | 1.22E-02 | -1.181 |
| hsa-miR-4632-5p | -2.071 | -2.450 | -1.692 | 4.686 | -3.839 | 8.78E-04 | 1.27E-02 | -1.225 |
| hsa-mir-4634 | -1.431 | -1.712 | -1.151 | 2.074 | -3.791 | 9.88E-04 | 1.42E-02 | -1.341 |
| hsa-miR-769-3p | 1.407 | 1.017 | 1.797 | 1.434 | 3.782 | 1.01E-03 | 1.44E-02 | -1.361 |
| hsa-miR-532-5p | 2.404 | 2.050 | 2.758 | 6.485 | 3.766 | 1.05E-03 | 1.50E-02 | -1.401 |
| hsa-miR-502-5p | 1.160 | 0.701 | 1.619 | 1.470 | 3.754 | 1.08E-03 | 1.54E-02 | -1.429 |
| hsa-miR-16-5p | 1.807 | 1.074 | 2.540 | 12.355 | 3.746 | 1.10E-03 | 1.56E-02 | -1.447 |
| hsa-miR-487b-3p | 1.636 | 1.103 | 2.169 | 1.706 | 3.728 | 1.15E-03 | 1.62E-02 | -1.490 |
| hsa-miR-1247-3p | 2.161 | 0.961 | 3.361 | 2.361 | 3.726 | 1.16E-03 | 1.62E-02 | -1.494 |
| hsa-miR-589-5p | 1.222 | 0.779 | 1.665 | 1.678 | 3.712 | 1.20E-03 | 1.68E-02 | -1.529 |
| hsa-mir-3656 | -1.155 | -1.473 | -0.837 | 2.285 | -3.705 | 1.22E-03 | 1.70E-02 | -1.545 |
| hsa-miR-28-5p | 2.692 | 1.773 | 3.612 | 6.189 | 3.700 | 1.23E-03 | 1.71E-02 | -1.556 |
| hsa-miR-3180 | -2.237 | -3.356 | -1.117 | 2.812 | -3.699 | 1.24E-03 | 1.71E-02 | -1.560 |
| hsa-miR-30d-5p | 2.060 | 1.304 | 2.815 | 6.029 | 3.681 | 1.29E-03 | 1.76E-02 | -1.600 |
| hsa-let-7f-5p | 2.756 | 2.350 | 3.161 | 5.728 | 3.681 | 1.29E-03 | 1.76E-02 | -1.602 |
| hsa-miR-92a-1-5p | 1.199 | 0.956 | 1.442 | 1.866 | 3.673 | 1.32E-03 | 1.79E-02 | -1.620 |
| hsa-miR-339-5p | 2.254 | 0.806 | 3.703 | 5.782 | 3.673 | 1.32E-03 | 1.79E-02 | -1.621 |
| hsa-miR-6813-5p | 2.370 | 2.028 | 2.712 | 2.662 | 3.652 | 1.38E-03 | 1.87E-02 | -1.670 |
| hsa-miR-1238-5p | -1.172 | -1.567 | -0.777 | 1.441 | -3.642 | 1.42E-03 | 1.91E-02 | -1.694 |
| hsa-miR-423-3p | 2.572 | 1.629 | 3.514 | 7.568 | 3.629 | 1.47E-03 | 1.96E-02 | -1.725 |
| hsa-let-7i-3p | 1.145 | 0.327 | 1.963 | 1.475 | 3.619 | 1.50E-03 | 1.99E-02 | -1.747 |
| hsa-miR-4695-5p | -1.390 | -2.756 | -0.024 | 5.211 | -3.617 | 1.51E-03 | 2.00E-02 | -1.753 |
| hsa-miR-6794-5p | -1.236 | -1.532 | -0.940 | 4.897 | -3.596 | 1.59E-03 | 2.08E-02 | -1.802 |
| hsa-miR-6798-5p | -2.013 | -2.343 | -1.683 | 3.898 | -3.566 | 1.71E-03 | 2.21E-02 | -1.872 |
| hsa-mir-4466 | -1.365 | -1.685 | -1.044 | 1.720 | -3.557 | 1.74E-03 | 2.26E-02 | -1.894 |
| hsa-miR-92b-5p | -1.966 | -3.094 | -0.839 | 4.344 | -3.529 | 1.87E-03 | 2.40E-02 | -1.960 |
| hsa-miR-6723-5p | -2.827 | -3.344 | -2.309 | 3.222 | -3.506 | 1.97E-03 | 2.51E-02 | -2.013 |
| hsa-miR-409-3p | 1.034 | 0.706 | 1.363 | 1.610 | 3.444 | 2.29E-03 | 2.84E-02 | -2.157 |
| hsa-mir-500b | 1.178 | 0.823 | 1.534 | 1.287 | 3.410 | 2.48E-03 | 3.05E-02 | -2.235 |
| hsa-miR-455-3p | -1.701 | -2.043 | -1.359 | 7.484 | -3.346 | 2.89E-03 | 3.52E-02 | -2.383 |
| hsa-miR-598-5p | -1.320 | -1.828 | -0.812 | 2.457 | -3.344 | 2.90E-03 | 3.53E-02 | -2.387 |
| hsa-miR-361-5p | 2.533 | 2.238 | 2.827 | 8.372 | 3.336 | 2.96E-03 | 3.58E-02 | -2.406 |
| hsa-miR-3607-5p | 1.060 | 0.325 | 1.794 | 1.540 | 3.314 | 3.12E-03 | 3.74E-02 | -2.457 |
| hsa-miR-4433-3p | 1.489 | 1.276 | 1.702 | 3.147 | 3.301 | 3.22E-03 | 3.83E-02 | -2.487 |
| hsa-miR-30b-5p | 1.872 | 1.533 | 2.212 | 6.189 | 3.234 | 3.78E-03 | 4.42E-02 | -2.641 |
| hsa-miR-6726-5p | 1.721 | 0.527 | 2.916 | 2.077 | 3.215 | 3.95E-03 | 4.60E-02 | -2.683 |
| hsa-miR-1303 | 1.009 | 0.756 | 1.261 | 1.424 | 3.211 | 3.99E-03 | 4.63E-02 | -2.693 |
| hsa-let-7d-3p | 1.765 | 1.417 | 2.112 | 3.517 | 3.207 | 4.02E-03 | 4.64E-02 | -2.701 |
| hsa-miR-664a-5p | 1.006 | 0.696 | 1.316 | 1.730 | 3.205 | 4.05E-03 | 4.65E-02 | -2.706 |
| hsa-miR-378i | 1.701 | 1.357 | 2.044 | 5.924 | 3.191 | 4.19E-03 | 4.73E-02 | -2.739 |
| hsa-miR-185-5p | 1.283 | 0.891 | 1.674 | 9.471 | 3.173 | 4.36E-03 | 4.90E-02 | -2.777 |
| hsa-miR-361-3p | 1.148 | 0.447 | 1.848 | 1.397 | 3.169 | 4.40E-03 | 4.93E-02 | -2.786 |

**Supplemental Table S5.** Differentially expressed miRNAs between the control group and MI patients in the nonclassical monocyte subset.

| gene | logFC | CI.L | CI.R | AveExpr | t | P.Value | adj.P.Val | B |
| --- | --- | --- | --- | --- | --- | --- | --- | --- |
| hsa-miR-378c | 3.981 | 3.396 | 4.567 | 7.408 | 12.686 | 1.16E-11 | 5.15E-08 | 15.170 |
| hsa-miR-378a-3p | 2.937 | 1.731 | 4.143 | 9.439 | 10.457 | 4.72E-10 | 1.04E-06 | 12.252 |
| hsa-miR-378f | 4.736 | 3.679 | 5.794 | 6.269 | 7.613 | 1.24E-07 | 1.83E-04 | 7.496 |
| hsa-miR-378i | 3.977 | 3.633 | 4.320 | 5.924 | 7.053 | 4.20E-07 | 4.64E-04 | 6.410 |
| hsa-miR-422a | 4.028 | 3.647 | 4.409 | 5.946 | 6.535 | 1.35E-06 | 1.19E-03 | 5.361 |
| hsa-miR-26a-5p | 1.585 | -0.009 | 3.179 | 11.562 | 5.977 | 4.92E-06 | 3.25E-03 | 4.184 |
| hsa-miR-532-5p | 4.002 | 3.689 | 4.315 | 6.485 | 5.927 | 5.53E-06 | 3.25E-03 | 4.078 |
| hsa-miR-191-5p | 1.309 | 1.005 | 1.613 | 11.970 | 5.901 | 5.88E-06 | 3.25E-03 | 4.022 |
| hsa-miR-93-5p | 1.452 | 1.087 | 1.817 | 9.831 | 5.689 | 9.72E-06 | 4.77E-03 | 3.563 |
| hsa-miR-6845-5p | -1.531 | -2.740 | -0.323 | 1.663 | -5.617 | 1.15E-05 | 5.11E-03 | 3.404 |
| hsa-miR-345-5p | 3.293 | 1.652 | 4.934 | 6.173 | 5.558 | 1.33E-05 | 5.33E-03 | 3.277 |
| hsa-miR-30d-5p | 3.217 | 2.614 | 3.821 | 6.029 | 5.437 | 1.77E-05 | 6.54E-03 | 3.011 |
| hsa-miR-4725-3p | -1.680 | -2.555 | -0.804 | 1.605 | -5.317 | 2.37E-05 | 8.07E-03 | 2.744 |
| hsa-miR-324-5p | 2.845 | 1.943 | 3.747 | 4.738 | 5.133 | 3.70E-05 | 1.10E-02 | 2.334 |
| hsa-miR-185-5p | 2.065 | 0.417 | 3.713 | 9.471 | 4.831 | 7.72E-05 | 2.02E-02 | 1.657 |
| hsa-miR-30b-5p | 2.957 | 2.528 | 3.385 | 6.189 | 4.828 | 7.78E-05 | 2.02E-02 | 1.650 |
| hsa-miR-4750-5p | -2.334 | -2.963 | -1.705 | 2.366 | -4.698 | 1.07E-04 | 2.63E-02 | 1.356 |
| hsa-miR-103a-3p | 1.504 | 1.137 | 1.872 | 12.174 | 4.617 | 1.30E-04 | 3.04E-02 | 1.173 |
| hsa-miR-140-3p | 1.479 | 0.703 | 2.254 | 9.107 | 4.554 | 1.52E-04 | 3.36E-02 | 1.032 |
| hsa-miR-652-3p | 2.919 | 1.201 | 4.637 | 7.243 | 4.504 | 1.72E-04 | 3.63E-02 | 0.917 |
| hsa-miR-339-5p | 2.832 | 2.484 | 3.179 | 5.782 | 4.362 | 2.44E-04 | 4.91E-02 | 0.595 |

**Supplemental Table S6.** Differentially expressed miRNAs between CAD patients and the control group in the classical monocyte subset.

| gene | logFC | CI.L | CI.R | AveExpr | t | P.Value | adj.P.Val | B |
| --- | --- | --- | --- | --- | --- | --- | --- | --- |
| hsa-miR-1307-5p | 2.151 | 1.373 | 2.929 | 1.642 | 7.651 | 1.14E-07 | 3.53E-04 | 7.701 |
| hsa-miR-301a-3p | 2.126 | 1.839 | 2.414 | 2.648 | 7.426 | 1.86E-07 | 3.53E-04 | 7.258 |
| hsa-miR-340-5p | 1.077 | 0.540 | 1.615 | 1.362 | 7.308 | 2.40E-07 | 3.53E-04 | 7.024 |
| hsa-miR-30e-3p | 2.384 | 0.807 | 3.961 | 2.252 | 7.034 | 4.39E-07 | 4.85E-04 | 6.468 |
| hsa-miR-148a-3p | 2.401 | 2.122 | 2.680 | 1.615 | 6.755 | 8.19E-07 | 6.15E-04 | 5.892 |
| hsa-miR-194-5p | 2.122 | 1.773 | 2.472 | 2.567 | 6.740 | 8.46E-07 | 6.15E-04 | 5.862 |
| hsa-miR-454-3p | 1.734 | 0.841 | 2.626 | 1.604 | 6.678 | 9.74E-07 | 6.15E-04 | 5.731 |
| hsa-miR-1226-3p | -2.097 | -2.334 | -1.861 | 1.818 | -6.562 | 1.27E-06 | 6.27E-04 | 5.488 |
| hsa-miR-542-5p | 1.753 | 1.397 | 2.110 | 1.528 | 6.524 | 1.38E-06 | 6.27E-04 | 5.407 |
| hsa-miR-125b-5p | 1.993 | 0.886 | 3.100 | 2.149 | 6.513 | 1.42E-06 | 6.27E-04 | 5.383 |
| hsa-mir-6503 | 1.267 | 0.335 | 2.199 | 2.260 | 6.278 | 2.44E-06 | 9.79E-04 | 4.881 |
| hsa-miR-199a-5p | 2.057 | 1.346 | 2.769 | 1.724 | 6.164 | 3.18E-06 | 1.17E-03 | 4.634 |
| hsa-miR-199b-5p | 1.505 | -0.007 | 3.018 | 1.512 | 6.032 | 4.32E-06 | 1.38E-03 | 4.346 |
| hsa-miR-30a-5p | 2.010 | 1.456 | 2.564 | 1.977 | 6.026 | 4.38E-06 | 1.38E-03 | 4.334 |
| hsa-miR-17-3p | 1.671 | 0.208 | 3.133 | 2.689 | 5.999 | 4.67E-06 | 1.38E-03 | 4.274 |
| hsa-miR-103a-2-5p | 1.557 | 1.164 | 1.950 | 1.654 | 5.600 | 1.20E-05 | 3.32E-03 | 3.392 |
| hsa-miR-652-5p | 1.238 | 0.661 | 1.816 | 2.208 | 5.541 | 1.38E-05 | 3.60E-03 | 3.258 |
| hsa-miR-326 | 2.201 | 1.230 | 3.171 | 2.013 | 5.485 | 1.58E-05 | 3.89E-03 | 3.133 |
| hsa-miR-4306 | 2.354 | 2.033 | 2.675 | 2.253 | 5.423 | 1.84E-05 | 4.27E-03 | 2.994 |
| hsa-miR-130a-3p | 2.629 | 1.801 | 3.457 | 3.122 | 5.248 | 2.80E-05 | 6.19E-03 | 2.598 |
| hsa-miR-19a-3p | 2.189 | 1.854 | 2.524 | 2.626 | 5.084 | 4.16E-05 | 8.77E-03 | 2.226 |
| hsa-miR-4685-5p | 1.016 | 0.271 | 1.761 | 1.705 | 4.865 | 7.11E-05 | 1.37E-02 | 1.724 |
| hsa-miR-21-3p | 1.733 | 1.396 | 2.070 | 2.149 | 4.818 | 7.97E-05 | 1.41E-02 | 1.616 |
| hsa-miR-1185-1-3p | 1.286 | 0.123 | 2.449 | 1.918 | 4.776 | 8.83E-05 | 1.50E-02 | 1.520 |
| hsa-miR-4286 | 1.203 | 0.154 | 2.251 | 2.813 | 4.755 | 9.29E-05 | 1.52E-02 | 1.472 |
| hsa-miR-660-3p | 1.363 | 0.642 | 2.085 | 1.332 | 4.615 | 1.31E-04 | 1.93E-02 | 1.150 |
| hsa-miR-7162-3p | -1.985 | -2.514 | -1.457 | 2.687 | -4.592 | 1.39E-04 | 1.96E-02 | 1.095 |
| hsa-miR-142-5p | 1.631 | 1.312 | 1.950 | 1.735 | 4.583 | 1.42E-04 | 1.96E-02 | 1.075 |
| hsa-miR-660-5p | 1.638 | 0.528 | 2.747 | 4.069 | 4.530 | 1.61E-04 | 2.16E-02 | 0.954 |
| hsa-miR-505-3p | 1.490 | 0.686 | 2.294 | 1.852 | 4.420 | 2.11E-04 | 2.75E-02 | 0.700 |
| hsa-miR-148b-3p | 1.756 | 1.441 | 2.072 | 2.721 | 4.366 | 2.42E-04 | 3.05E-02 | 0.575 |
| hsa-miR-1185-2-3p | 1.361 | 1.044 | 1.678 | 1.869 | 4.340 | 2.58E-04 | 3.17E-02 | 0.515 |
| hsa-miR-143-3p | 1.445 | -0.061 | 2.952 | 2.212 | 4.202 | 3.61E-04 | 4.13E-02 | 0.198 |
| hsa-miR-6775-5p | -1.029 | -1.815 | -0.243 | 7.194 | -4.198 | 3.64E-04 | 4.13E-02 | 0.189 |
